# Effects of pitch and musical sounds on body-representations when moving with sound

**DOI:** 10.1038/s41598-022-06210-x

**Published:** 2022-02-17

**Authors:** Judith Ley-Flores, Eslam Alshami, Aneesha Singh, Frédéric Bevilacqua, Nadia Bianchi-Berthouze, Ophelia Deroy, Ana Tajadura-Jiménez

**Affiliations:** 1 DEI Interactive Systems Group, Department of Computer Science and Engineering, Universidad Carlos III de Madrid, Avenida de la Universidad 30, 28911 Leganés, Madrid, Spain; 2 UCL Interaction Centre (UCLIC), University College London, University of London, London, UK; 3 IRCAM Institut de Recherche et Coordination Acoustique Musique, Sorbonne Université, Paris, France; 4 Faculty of Philosophy, Philosophy of Science and Religious Studies, Ludwig-Maximilians-Universität München, Munich, Germany; 5 Munich Center for Neuroscience, Ludwig-Maximilians-Universität München, Munich, Germany; 6 School of Advanced Study, University of London, London, UK

**Keywords:** Cognitive neuroscience, Perception

## Abstract

The effects of music on bodily movement and feelings, such as when people are dancing or engaged in physical activity, are well-documented—people may move in response to the sound cues, feel powerful, less tired. How sounds and bodily movements relate to create such effects? Here we deconstruct the problem and investigate how different auditory features affect people’s body-representation and feelings even when paired with the same movement. In three experiments, participants executed a simple arm raise synchronised with changing pitch in simple tones (Experiment 1), rich musical sounds (Experiment 2) and within different frequency ranges (Experiment 3), while we recorded indirect and direct measures on their movement, body-representations and feelings. Changes in pitch influenced people’s general emotional state as well as the various bodily dimensions investigated—movement, proprioceptive awareness and feelings about one’s body and movement. Adding harmonic content amplified the differences between ascending and descending sounds, while shifting the absolute frequency range had a general effect on movement amplitude, bodily feelings and emotional state. These results provide new insights in the role of auditory and musical features in dance and exercise, and have implications for the design of sound-based applications supporting movement expression, physical activity, or rehabilitation.

## Introduction

“To dance is to be out of yourself. Larger, more beautiful, more powerful… This is power, it is glory on earth and it is yours for the taking”, wrote the renowned dancer and choreographer Agnes De Mille^1^. Such passionate defences of dance are not rare, but can they be taken literally? Experimental evidence stresses the benefits of dance, especially for mobility, body acceptance and self-awareness^2,3^, yet whether dance can affect how large or powerful one feels would suggest that it acts on the flexibility of one’s body representation. If it does, according to which mechanisms would this occur?

How we feel about our body but also how we represent it mentally can change through time because of external signals. Body-representations^4^ are indeed very malleable^5–7^ and not just updated through gradual periods of development, but also on the fly according to incoming sensory information^8,9^ (see review by Azañón et al.^10^), including sounds^11–13^. Because body-representations are not unified, but processed in a spatiotemporally distributed way in the brain, we should distinguish representations of body appearance known as “body image” (e.g., size or weight^5,6^) from representations of body parts position and body kinematics, on which people rely whenever they move, reach for objects, or manipulate tools, known as “body schema”^14–16^. The latter component can also be subconscious and shape our body movement and interactions with the surrounding environment (e.g.,^14,17^). Together though, the various components of body-representations influence how people subjectively feel about their body and its physical capabilities (for instance, feeling light or strong) and about their movement (for instance, finding it easier or more comfortable) and this can in turn interact with one’s emotional state.

To investigate whether dance affects body representation, we propose to start with a simpler model and abstract away from the wide diversity of genres, forms and practices of dance which exist across cultures and evolving sub-cultures. Looking at dance at an abstract and basic level, we focus on its multisensory and performative aspect at the scale of a basic movement: What is the effect of sounds or music when they accompany a bodily movement or when they are played in the background? Many types of dances rely on external sounds, in addition to the natural sounds produced by footsteps or clapping of the dancers themselves, and aim at synchronising these sounds with bodily movements^18^. The synchrony between the sounds and the signals arising from the moving body, as well as cross modal correspondence can explain how dance comes to affect “bodily movement”—making us, for instance, move faster or slower when pitch increases. They can also explain changes in “proprioceptive awareness”—making us for instance more or less aware of where our limbs are in space, and “bodily image and feelings”—making us feel for instance lighter, heavier, smaller, or taller, as well as more comfortable or in control.

Which of these various aspects of bodily representations and feelings are then most influenced when one moves in synchrony with sounds? While we know more about how beats influence movements rhythmically performed through time^19^, do basic auditory and musical features such as pitch changes, loudness, timber and frequency range also affect the body during a single movement execution?

To tackle this experimental challenge, we isolated and measured the effects of sounds played in synchrony with a basic bodily movement: We asked participants to execute a simple arm movement, raising the arm sideways, while listening either to pure tones varying in pitch (Experiment 1) or to musical sounds, with richer timber (i.e., containing several harmonics), varying in loudness (Experiment 2). Using indirect and direct methods, we assessed how the sounds affected their awareness of their arm position, their movement and their bodily and emotional feelings. In Experiment 3, we asked whether the effects of pitch changes were relative, and depended only on the direction of change, or were dependent also on the absolute range of frequencies used. Altogether, the three experiments provide more precise guidelines to select sounds and music more effectively in therapeutic or well-being interventions involving physical activity.

We were interested in seeing whether a change in *pitch played a pivotal role* in explaining the bodily effects of sounds listened during body movement or any forms of physical activity, through a multisensory binding analogous to the one found in other proprioceptive illusions such as the rubber hand illusion^7^. To test this hypothesis, we expected the effects to occur only when the sounds, in the three experiments, were presented in synchrony with the prescribed movement. The previous literature gives us good reasons to expect that pitch change would affect all three aspects of motion, bodily awareness and bodily feelings in specific ways (see Table 1), but each experiment enables us to address additional questions. In *Experiment 1*, we could compare whether the changes in movement, proprioceptive awareness and bodily feelings would all be equally sensitive to the congruence between pitch and motion direction. In *Experiment 2*, we could assess whether and how a richer musical timbre (i.e., a richer spectrum with several harmonics and dynamic changes in loudness) would enhance, diminish the effect of pitch on movement, awareness and bodily feelings, or influence them in other manners. Because harmonics are shown to be overall more pleasant to listen to, we expected an increase in the bodily feelings, in the positive direction, but not in the other effects. However, if crossmodal correspondences between pitch and upward/downward space are emotionally mediated (^20^; see also^21,22^ for reviews) then we could also see a more general increase in all three effects. In addition, because the dynamic changes in loudness often present in music interact with the perception of pitch^23–26^, and can also elicit impressions of changes in spatial distance^27,28^ a richer musical timbre may modulate (either maximize or diminish) the effects observed for pure tones.

**Table 1:** Specific predictions/replications for the robust effect of pitch changes across all the experiments.

Dimension	Predicted effects of raising versus descending pitch	Effects reported in previous literature (reference)
*Bodily movement*	*Movement parameters*: A sound increasing or decreasing in pitch accompanying the participant’s arm movement will respectively increase/decrease participants’ arm vertical movement amplitude and its acceleration/velocity, as if the sound would “pull up”/“push down” the body	*Perceptions of motion for objects outside the body*: Dynamic changes in pitch elicit perceptions of changes in height, size and motion along the vertical plane (^29^; see review by^30^). Associations of tonal sounds rising in pitch with motion upwards have been also found in gestural depictions of sounds^31^ *Effects of harmonic content (stability of musical sounds) on bodily movement*: Musically resolved (i.e., ending on a perfect or harmonically stable cadence) vs. unresolved (i.e., ending on an imperfect or harmonically unstable cadence) sonifications accompanying arm raise movements lead people to increase the movement amplitude and stretch for longer, potentially due to musical expectation^32^ *Effects of frequency range on bodily movement*: Shifting the pitch of walking sounds to make the sounds consistent with having a heavier or lighter body results in changes in the leg movement acceleration and stance time^12,33^
*Proprioceptive awareness*	*Accuracy of and confidence in perceived final position*: A sound increasing or decreasing in pitch accompanying the participant’s arm movement will lead participants to be less accurate and become less confident about their arm position, as a result of sound interfering with proprioception	*Sound influence on accuracy of perceived object position*: This is illustrated by literature on the ventriloquism illusion, by which people mislocalize the source of speech sounds when incongruent visual cues are synchronously presented (e.g.,^34^) *Sound influence on confidence in perceptual performance*: Literature on the McGurk effect^35^, by which people misperceive incongruent visual and auditory cues, shows not only a decrease in perceptual accuracy, but also effects on the subjective confidence in perceptual performance^36^ *Illusory body extension potentially driven by sound influences on proprioception*: When brief sounds rising in pitch are paired and presented synchronously with the action of oneself pulling on one’s occluded fingertip can lead to participants feel and estimate their finger to be longer^13^ suggesting influences of sound on proprioception. This illusion was replicated both in adults and pre-school children for passive finger pulling^37^ *Influence of harmonic stability of musical sounds on perceived body position*: Musically resolved or unresolved sonifications accompanying squat movements impact on the perceived depth of the squat^38^ *Influence of movement sonification on movement variability*: Movement sonification can induce higher movement variability for both musicians and non-musicians when starting to learn a new movement sequence, while it is reduced later when the movement is mastered. It has been hypothesized that the sound feedback provokes a change of attentional focus that perturbates proprioceptive awareness^39^
*Bodily and emotional feelings*	*Feelings about one’s body, the movement, and emotional state*: A sound increasing or decreasing in pitch accompanying the participant’s arm movement will impact on how people feel “about their body” (e.g., weight or speed) and “about their movement” (e.g., ease, comfort). Sounds increasing vs. decreasing in pitch will enhance the emotional state, making people feel happier, more excited and motivated	*Perceptions of size for objects outside the body*: Pitch is associated to physical size; static high and low pitches are respectively congruent with smaller and larger sizes^40–44^ *Effect of pitch (frequency range) on perceived body size and feelings*: Shifting the pitch of walking sounds to higher frequencies makes people experience their body as being slimmer and lighter than usual, as well as quicker and happier, while the opposite is true for lower pitch sounds^12^. With high-frequency footsteps sounds people find step-up exercises less difficult and feel less tired^33^ *Effects of change on pitch on bodily and emotional feelings*: In a qualitative study people reported that a sound rising in pitch paired with bodily movement induces pleasantness and feelings of movement fluidity and body lightness and flexibility^45^ Sequences of tonal beeps or notes changing in musical pitch and sonifying trunk movement during forward reach exercises help to build confidence and motivate people with chronic pain to move despite pain and fear of injury^46–48^ *Effects of harmonic content*: Musically resolved sonifications accompanying stretching and squat movements increase feelings of reward and achievement, as well as motivation to continue the movement^32,38,49^

Finally, *Experiment 3* could assess whether the relative direction of pitch change was all that mattered, or whether the absolute frequency range also modulated and explained the effects. Here the literature on crossmodal correspondences (see^50^) gives us reasons to predict that the relative change was all that mattered, notably for more automatic effects on motion and proprioception; for instance, in terms of mapping with changes in spatial elevation, the absolute frequency range is less significant than the direction of the frequency change^51^. But other previous results (see Table 1) made us expect that the absolute frequency range would affect bodily feelings, with higher frequencies making people, for instance, feel significantly lighter than lower frequencies^12,33^.

## Methods

Here, we report three experiments in which we asked participants to raise their right arm to reach a pre-trained position. We evaluated the effects of different sounds on participants’ bodily movement (i.e., lifting amplitude, velocity, acceleration, time) and on their proprioceptive awareness, measured in terms of accuracy of final arm position (i.e., elevation angle of the arm), as well as on the confidence on having reached that position, as changes in pitch may lead to the illusion of vertical displacement of one’s arm as if this was being “pulled up” or “pushed down” by the sound. We further investigated the effects on body-representations in terms of subjective feelings about one’s body (e.g., lightness, strength) and the movement (e.g., movement ease, capability to perform the movement), as well as the effects on emotional state which may accompany these changes.

### Experiment 1: effects of pitch change (sound direction)

#### Participants

Twenty-five participants took part (Age: Mean = 27.68 years, SD = 5.83, Range = 20–39; 11 female, 14 male). In all experiments reported here participants gave their informed consent prior to their inclusion in the studies and the study was conducted in accordance with the ethical standards laid down in the 1964 Declaration of Helsinki. Experiments 1 and 2 were approved by the local ethics committee at Universidad Carlos III de Madrid (UC3M). The same participants took part both in Experiments 1 and 2 in exchange for 10 euros. Participants took part in Experiment 2 after having completed Experiment 1.

#### Apparatus

A wearable self-locking band equipped with a hand-sewn cloth pocket containing a wireless emitter (BITalino R-IoT embedding a 9-axis Inertial Motion Unit (IMU) digitized at 16 bits) was used, based on^52,53^. The band wirelessly transmits data through WiFi using the OSC protocol to a computer running Max/MSP (Cycling’74) and can detect the start of the movement and trigger then a sound to accompany the movement (i.e., sonification). The device is calibrated to the range of movement to be sonified for a specific person through a graphical user interface. To do so, the configuration of the arm at the start (minimum movement angle) and at the end of the movement (maximum movement angle) are registered. The software also records the movement data for posterior off-line analysis. The sound was fed back to participants through wired headphones (Sennheiser HD 2.30G).

#### Stimuli

**Figure 1:**
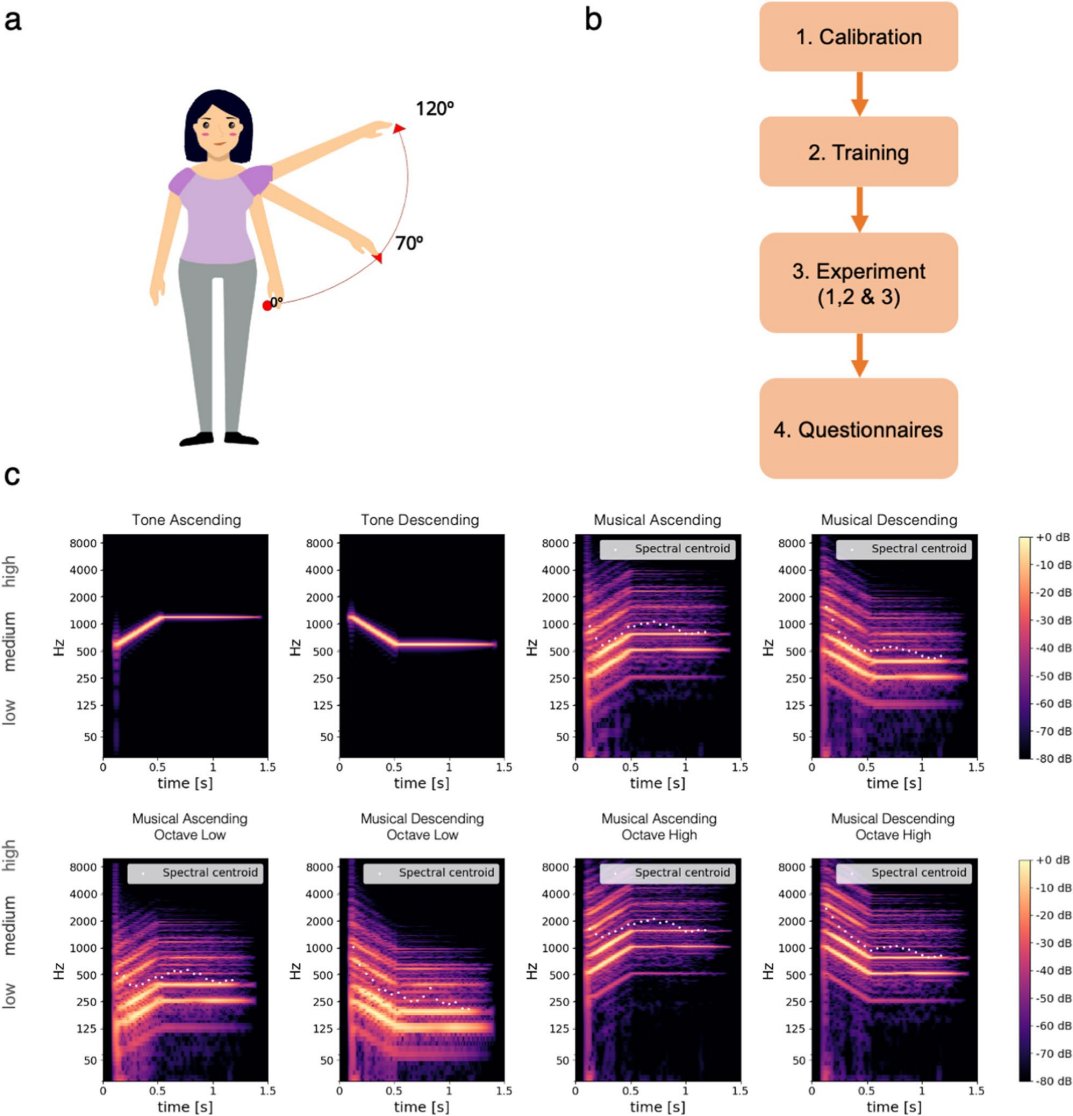
Side arm raise movement, graphical representation of the experimental procedure and spectra of the different Tones and Musical Sounds used in the experiments. (**a**) Across conditions, participants were requested to raise their arm from 0° to 70° (Position 1) or from 0° to 120° (Position 2). (**b**) The experimental procedure consisted of four phases: calibration, training, experiment (with behavioral data acquisition) and questionnaires. (**c**) The different plots from top-left to bottom-right correspond to the following stimuli: “Tone_up”, “Tone_down”, “Musical_up”, “Musical_ down”, “Musical_up_Low_pitch”, “Musical_down_Low_pitch”, “Musical_up_High_pitch”, “Musical_down_High_ pitch” (see the summary of experimental conditions and factors studied with these sounds in Table 2).

Three auditory stimuli were used, drawing on previous studies by^13,37^. They consisted of pure tones (1300-ms duration and 44.1-kHz sample rate) with ascending (“Tone_up”, 600 to 1200 Hz), descending (“Tone_down” 1200 to 600 Hz) or constant (“Tone_constant”, 600 Hz) frequency (see Fig. 1). The pitch change occurs during 500 ms, followed by a sustained part of 500 ms, and a decay of 300 ms. Note that the frequency range employed slightly differed from the one used by^13^ (i.e., 700–1200 Hz). The choice of 600 Hz, instead of 700 Hz, was made to ensure a full octave in the ascending and descending sounds, providing a target sound that appears natural from a musical point of view as going up or down a full scale. Further, note that the constant sound was included as a “control” or reference condition with which to compare the effect of the sounds changing in pitch (as in^13,37^). This was preferred to a “no sound” condition, as it allowed controlling for the effect of simply listening to a sound (see other studies using similar control condition^28,45^).

#### Movement exercise

A side arm raise exercise was chosen, as this is a basic exercise that involves the challenge of raising the arm to different angles and strengthening the upper arms (see Fig. 1). This gesture is part of many dance sequences and it is also an exercise that is part of the “general warm up” or toning routines of many programs or guidelines oriented toward dance or general physical activity (e.g., guidelines for becoming more physically active^54,55^).

#### Experimental procedure

The experiment was conducted in a quiet room and consisted of four phases: calibration, training, experiment and questionnaires, as detailed below (see Fig. 1b). The full procedure took approximately 25 min.

##### Calibration

Firstly, participants were asked to stand with their back against a whiteboard. Secondly, the experimenter drew on the whiteboard a point at shoulder height while the participant held her right arm in a soldier's position (this was marked as the movement start position i.e., angle of 0°). Thirdly, three lines were drawn to indicate the initial position (angle of 0°) and the angles of 70° and 120° that indicate position 1 and 2 respectively during the experiments (See Fig. 1a). Note that two positions, rather than only one (as in^56^), were chosen to avoid habituation and increase participants’ concentration on their perceptions of their hand position. The position of the arm at 0° (minimum movement angle) and 120° (maximum movement angle) were registered by the Max/MSP software respectively with the values 0 and 1. This calibration was performed in order for the software to recognize these positions and to trigger a sound in the experimental trials when identifying that the arm left the minimum movement angle (i.e., the 0° position).

##### Training

Participants were asked to lift their arms laterally five times to position 1 and five times to position 2, in the order indicated by the experimenter, and with their eyes opened. This allowed participants to practice their arm movement to reach both positions. Participants were then asked to close their eyes and lift their arms laterally five times to position 1 and five times to position 2, in the order indicated by the experimenter. No feedback sound was delivered. Further, the experimenter did not provide any feedback to participants on their performance.

##### Experiment

Participants were told that in each trial, with their eyes closed, they would be asked to lift their arm laterally to reach either position 1 or position 2, as indicated verbally by the experimenter at the start of the trial, as in the training phase. Once participants were indicated the target position, they initiated the movement when they felt like, and their movement onset triggered a sound that was irrelevant to the task (i.e., stimuli were not time-locked to the experimenter instruction, but to participants’ movement onset). In each trial, participants listened to one of the three sounds (“Tone_up”, “Tone_down”, or “Tone_constant”). Note that even if participants returned to the start position after each arm raise, the sound was only triggered by the upwards movement. Each sound was presented ten times (as in^13^), five times per position (30 arm lifts in total). The different combinations of sounds and positions were presented randomly to minimise order bias. See “Table 2, Summary of the experimental conditions”.

**Table 2:** Summary of the experimental conditions (ordered randomly), factors and number of repetitions and trials in Experiments 1, 2 and 3.

Experiment	Sound condition	Sound direction	Sound timbre	Sound frequency range	Repetitions/total nr. trials
Experiment 1	Tone_constant	Constant	Tone	Medium	10 per condition (5 per position)/30
	Tone_up	Up			
	Tone_down	Down			
Experiment 2	Tone_up	Up	Tone	Medium	10 per condition (5 per position)/40
	Tone_down	Down			
	Musical_up	Up	Musical sound		
	Musical_down	Down			
Experiment 3	Musical_up_Low_pitch	Up	Musical sound	Low–medium	10 per condition/40
	Musical_down_Low_pitch	Down		Medium–low	
	Musical_up_High_pitch	Up		Medium–high	
	Musical_down_High_pitch	Down		High–medium	

##### Questionnaire

At the end of the 30 experiment trials, participants were asked to repeat the arm lift task while listening to a tone for six more trials, two trials for each sound condition, and to complete a questionnaire for each sound condition (similarly to the procedure followed in^13^). For each sound condition, participants repeated two arm lift trials, of which one trial corresponded to Position 1 and the other trial to Position 2, with the presentation order randomized across participants. After each arm lift, we asked participants how confident they were of having reached the requested position with the current sound (Survey section 1—Confidence). Participants were then asked to complete a self-report of their body feelings when performing the task with that sound (Survey section 2—Body feelings). This survey is detailed in the next section. Participants repeated the survey procedure for the three sound conditions (their order presentation was randomized).

#### Measures

To monitor changes in bodily movement, task confidence (i.e., confidence in perceived final position, related to sound influences in proprioceptive awareness), bodily and emotional feelings across the different sound conditions the following measures were used:

##### Bodily movement

The movement sensor data were used to quantify changes in the reached angle and in the movement dynamics (time, velocity, and acceleration). In particular, the following parameters were extracted using MATLAB software (based on^52^): maximum (peak) and mean angle; time from minimum to maximum position (time up) and from maximum to minimum position (time down); mean angular velocity from minimum to maximum position (velocity up) and from maximum to minimum position (velocity down); and maximum linear acceleration from minimum to maximum position (acc up) and from maximum to minimum position (acc down).

##### Task confidence (confidence in perceived final position)

Explicit confidence (or certainty judgments) allows us to assess the reliability of perception across different decisions (e.g.,^57^); it relates to subjective estimates of being right rather than objective accuracy, and therefore falls within the field of metacognition. To assess the participants’ confidence in the perceived final position, Sect. 1 in the survey included a question about confidence reaching the requested position (i.e., position 1 or 2), “How confident were you with this sound that your arm was in the <position>?”, which was based on previous research assessing task confidence^57–59^. Participants answered using a 7-point Likert-type scale, ranging from 1: “not confident at all” to 7: “completely confident”.

##### Bodily and emotional feelings

Survey section 2 included 9 items (7-point Likert-type) and was developed based on the questionnaires used in related studies^12,33,52^. Three items related to how people *feel about their body during the exercise*—they began with “As I was doing the exercise, I felt…” and then ranged from “Light” to “Heavy” (Weight); “Slow” to “Quick” (Speed); “Not tired” to “Tired” (Tiredness). Four items related to their *feelings about the movement* and the endurance to perform the exercise: “As I was doing the exercise, I felt…” then ranged from “Not in control” to “In control” (Control) and from “Uncomfortable” to “Comfortable” (Comfort); “With this sound the exercise was…” then ranged from “Easy” to “Difficult” (Difficulty or ease); “I felt” then ranged from “Incapable” to “Capable” of performing the exercise (Capability). Another item assessed the emotional effects of the sounds heard on motivation to do the exercise (from “Did not motivate” to “Motivated me” to do the exercise (Motivation). Finally, we included an item related to felt agency over the heard sounds (ranging from “Not produced” to “Produced by me”; Agency), as previous studies have shown that if agency is disrupted (for instance, due to large discrepancies between modalities or delays between actions and sensory feedback) then sensory-induced body effects diminish (e.g.,^11,60^). Note that the repartition of items into the different categories (e.g., feelings about the body or feelings about the movement) was not made explicit to participants, and it is presented like this here to facilitate the assimilation of effects; some of the items could fall into two categories (e.g., “I felt uncomfortable” might be interpreted relative to the movement itself, or more generally about the body).

#### Data analyses

For *movement data*, for each of the parameters extracted data were first analyzed with separate repeated-Measures 3 × 2 × 5 analyses of variance (ANOVAs), suitable for continuous normal data, with within-subject factors Sound Direction (Ascending, Descending or Constant), Position (1 or 2), and Repetition (1 to 5). Given that there was no significant effect of the factor Repetition or interaction of Repetition with the other factors, data from the 5 repetitions for each condition were averaged and 3 × 2 ANOVAs were run with the factors Sound Direction and Position. Significant effects were followed by paired t-tests comparing the means obtained for the different conditions, which were corrected for multiple comparisons with the recommended Tukey method for comparing a family of estimates^61^.

For *questionnaire data*, to investigate on the Task confidence data the interaction between the factors Sound Direction (Ascending, Descending or Constant) and Position (1 or 2), we ran non-parametric ANOVAs on aligned rank transform (ART) data, suitable for ordinal data, using the R package ARTool^61^. Running ANOVAs allowed investigating the interaction between the factors Sound Direction and Position. For the data on Bodily and Emotional Feelings, we ran non-parametric ANOVAs on ART data with a single within-subject factor, Sound Direction. Significant main effects were followed by paired t-tests on the ART data, which were corrected for multiple comparisons (Tukey method). In addition, to compare self-reported confidence in the position with the actual task performance (i.e., measured position), we conducted Spearman correlation analyses for each of the conditions between the maximum angle (average of all repetitions for the condition) and the task confidence rating provided by the participant for that condition.

### Experiment 2: effects of harmonic content (sound direction and timbre)

#### Participants

Same participants that took part in Experiment 1. All participants performed Experiment 2 after having completed Experiment 1.

#### Apparatus

Same as in Experiment 1.

#### Stimuli

Four auditory stimuli were used. Two of the stimuli were ‘Tone_up’ and ‘Tone_down’ sounds employed in Experiment 1. We recall that the Tones were created using a single frequency whose pitch is varied one octave up or down (i.e., frequency being multiplied or divided by 2, respectively), as shown in Fig. 1c. The pitch change occurs during 500 ms, followed by a sustained part of 500 ms, and a decay of 300 ms. The other two stimuli consisted of musical sounds, “Musical_up” and “Musical_down”, designed with the same duration and pitch variation of one octave up or down, based on^52^. For both the Tone and Musical Sounds, the pitch change occurs during the first 500 ms, and then remains constant for 1 s (see Fig. 1c).

While the Tone sound spectrum is formed by a single frequency (sometimes referred as “pure” sound), the Musical Sound exhibits a rich spectrum and more complex energy enveloppe, formed by an attack peak at 200 ms, followed by a decay (300 ms), sustained part (500 ms), and release (300 ms). The Musical Sound is made of two notes, a fifth interval (e.g., C-G) that is considered in music theory as consonant and neutral. While we could have also used a single musical note, the choice of the consonant fifth interval was motivated to produce a higher contrast to the pure tone in terms of spectral richness, without adding any musical tension from a perception point of view. As shown in Fig. 1c, the musical sound spectrum is formed by several harmonics that span from 130 Hz to more than 6000 Hz. Importantly, the spectral centroid of the Musical Sound is comparable to the Tone frequency range (600–1200 Hz).

In summary, the main differences between the Tones and Musical Sounds reside in (1) the sound timbre given by the spectrum structure (harmonic content) and (2) the audio energy temporal envelope, with a stronger attack for the Musical Sound.

#### Exercise

Same as in Experiment 1.

#### Experimental procedure

Calibration and training phases were identical to those in Experiment 1. The experiment and questionnaire phases differed in that there were four instead of three sound conditions. In the experiment phase, each sound was presented ten times, five per position, as in Experiment 1 (40 arm lifts in total). The sounds were randomly ordered to minimise order bias. The full procedure took approximately 25 min.

#### Measures

Same as in Experiment 1.

#### Data analyses

For *movement data*, for each of the parameters extracted data were analyzed by conducting separate 2 × 2 × 2 × 5 ANOVAS with within-subject factors Sound Direction (ascending, descending), Timbre (Tone, Musical), Position (1 or 2) and Repetition (1 to 5). As in Experiment 1, there was no significant effect of the factor Repetition or interaction with the other factors. Therefore, data from the 5 repetitions for each condition were averaged and ANOVAs were run with the factors Sound Direction, Timbre and Position. Significant effects were followed by paired t-tests, which were corrected for multiple comparisons (Tukey method).

For *questionnaire data*, to investigate the interaction between the factors Sound Direction (Ascending, Descending) and Timbre (Tone, Musical), we ran non-parametric ANOVAs on ART data using ARTool. For the data on Task confidence, an additional factor of Position (1 or 2) was added to the ANOVAs. Significant interactions between factors were followed by interaction contrasts, which look at differences of differences, using the “testInteractions” function^62,63^, which is part of the R Phia module. The Holm method for p-value adjustment was used, as recommended.

As in Experiment 1, we conducted Spearman correlation analyses for each of the conditions between the maximum angle (average of all repetitions for the condition) and the task confidence rating provided by the participant for that condition.

### Experiment 3: effects of absolute frequency range (sound direction and sound frequency range)

#### Participants

Twenty participants took part (Age: Mean = 25.1 years, SD = 3.13, Range = 22–34; 9 females, 11 male). Experiment 3 was approved by the local ethics committees at UC3M and at University College London. Participants took part in exchange for a raffle, giving them an opportunity to win one of several Amazon vouchers (£30 × 3, £10 × 6).

#### Apparatus

Due to the Covid-19 Lockdown, participants were asked to use their own headphones and their own Android phones with a software application (Go-with-the-Flow-Moves@HOME, supporting Android 6.0 and superior versions) developed for research purposes. The design of the application was based on^47^. Using the accelerometer and gyroscope sensors, the application can detect the movement and calibrate the mobile to the range of the movement, similarly as in Experiment 1 and 2. Wearable arm straps were provided through postal services. Microsoft Teams software was used for the Experimenter to guide and interact with participants, closely monitoring the experiment. A Qualtrics survey was used to record responses to Likert-type questionnaire items.

#### Stimuli

Four auditory stimuli were used to explore the effect of the baseline pitch on actual and perceived motion (see Fig. 1c). These were variations of the “Musical_up” and “Musical_down” sounds employed in Experiment 2, in which the sound frequency range was shifted either one octave up or one octave down, as described here: “Musical_up” pitch shifted one octave down (“Musical_up_Low_pitch”), “Musical_up” pitch shifted one octave up (“Musical_up_High_pitch”), “Musical_down” pitch shifted one octave down (“Musical_down_Low_pitch”), “Musical_down” pitch shifted one octave up (“Musical_down_High_pitch”).

#### Exercise

Same as in Experiments 1 and 2, with the only difference that participants were asked to raise their arm until it reached a horizontal position (an angle of 90°, as in^56^). Note that, differently from Experiments 1 and 2, only one position was employed to reduce the experimental length due to time restrictions.

#### Experimental procedure

Participants were asked to be in a quiet room at home and to stand up during the experiment. No training or baseline phases were used. Participants were asked to raise their arms laterally to a 90° position while listening to one of four sounds (“Musical_up_Low_pitch”, “Musical_up_High_pitch”, “Musical_down_Low_pitch”, “Musical_down_High_pitch”). As in Experiments 1 and 2, the sound was only triggered by the upwards movement. Each sound was presented ten times (40 arm lifts in total). The sounds were randomly ordered to minimize order bias (See Table 2. Summary of the experimental procedure). At the end of the 40 experiment trials, participants were asked to repeat four additional arm lift trials, one for each sound condition, and after each sound presentation they filled in an online survey with 13 items asking about emotion and body feelings when performing the task with that sound. This survey is detailed in the next section. The full procedure took approximately 40 min.

#### Measures

As in Experiments 1 and 2, self-report and behavioral measures were collected to monitor changes across the different sound conditions. Note that, differently from Experiments 1 and 2, to reduce the experimental length due to time restrictions task confidence was not assessed, but additional items were added to the survey which allowed to further investigate the effects on bodily and emotional feelings.

##### Bodily movement

The movement sensor data recorded by the app were used to quantify changes in the maximum reached angle (peak angle) and in the movement dynamics (time, velocity, acceleration) of the upwards movement. Same parameters as in Experiments 1 and 2 (except for movement dynamics of the downwards movement, due to the app only tracking the upwards movement) were extracted using MATLAB software.

##### Bodily and emotional feelings

A survey with 13 items (Likert-type) was used to investigate how each sound affects the emotional and bodily feelings of participants during the lateral arm raises. The first 2 items corresponded with the two Self-Assessment Manikin graphical scales (9-point Likert-type response items) for valence and arousal^64^. Participants were asked to “select the figure that best represents how you felt during the single arm raise experience”. The first item ranged from “Unhappy, Negative” to “Happy, Positive” (Valence) and the second item ranged from “Unaroused, Calm” to “Aroused, Excited” (Arousal). The remaining items were 7-point Likert-type response items. 9 items were the same as those included in the survey in Experiments 1 and 2; the 2 additional items were the following: first item began with “As I was doing the exercise, I felt…” and then ranged from “Weak” to “Strong” (Strength). The second item began with “As I was doing the exercise, I felt my movement was…:” and then ranged from "Uncoordinated” to “Coordinated” (Coordination).

#### Data analyses

For *movement data*, for each of the parameters extracted data were analyzed by conducting separate 2 × 2 × 10 ANOVAS with within-subject factors Sound Direction (Ascending, Descending), Sound Frequency Range (High, Low), and Repetition (1 to 10). As in Experiments 1 and 2, there was no significant effect of the factor Repetition or interaction with the other factors. Therefore, data from the 10 repetitions for each condition were averaged and only the factors Sound Direction and Sound Frequency Range were considered in the analyses. Significant effects were followed by paired t-tests, which were corrected for multiple comparisons (Tukey method).

For *questionnaire data*, to investigate the interaction between the factors of Sound Direction (Ascending or Descending) and Sound Frequency Range (High or Low), we ran non-parametric ANOVAs on ART data using the “ARTool” package. Significant main effects were followed by paired t-tests, which were corrected for multiple comparisons (Tukey method).

## Results

The next three subsections describe the sound effects on bodily movement, proprioceptive awareness (task confidence and position accuracy) and bodily and emotional feelings observed across the three experiments. Table 3 summarizes all findings (for a more detailed summary of results see Supplementary Table S4). Median and range values for all questionnaire items in Experiments 1–3 are provided as Supplementary Material (note that medians are provided instead of means as they are more suitable for ordinal data as the data obtained using Likert scales^65^.

**Table 3:** Schematic summary of results on the effects across all the experiments (for a detailed summary of results see Supplementary Table S4).

Dimension		Predicted effects	Effects of pitch change	Effects of timbre (vs tone)	Effects of absolute frequency range
Bodily movement		Amplitude	✕	✕	✓
		Acceleration/velocity	✓	✓	✕
Proprioceptive awareness		Accuracy of final position	✕	✕	✓
		Confidence on perceived final position	✓	✓	Not assessed
Bodily and emotional feelings	Feelings about the body	Weight	✓	✓	✓
		Speed	✓	✕	✓
		Tiredness	✓	✓	✓
		Strength	✕	Not assessed	✕
	Feelings about the movement	Sense of control	✓	✕	✕
		Ease	✓	✓	✓
		Comfort	✓	✓	✕
		Capability	✓	✓	✕
		Coordination	✓	Not assessed	✕
	Emotional feelings	Motivation	✓	✓	✓
		Happiness	✓	Not assessed	✓
		Arousal	✕	Not assessed	✕

### Experiment 1: effects of pitch change (sound direction)

#### Effects on bodily movement

The analyses of the movement data showed a significant effect of the factor Position for most of the extracted parameters. As expected, for the condition where participants were asked to reach Position 2, the mean angle (F(1,24) = 217.83, *p* < 0.001, η^2^ = 0.90) and maximum angle (F(1,24) = 222.76, *p* < 0.001, η^2^ = 0.90) were higher. This effect of position was also reflected in longer “time down” (F(1,24) = 21.34, *p* < 0.001, η^2^ = 0.47), higher “velocity up” (F(1,24) = 148.70, *p* < 0.001, η^2^ = 0.86) and higher “velocity down” (F(1,24) = 57.32, *p* < 0.001, η^2^ = 0.70), higher “acceleration up” (F(1,24) = 67.49, *p* < 0.001, η^2^ = 0.74), and higher “acceleration down” (F(1,24) = 70.47, *p* < 0.001, η^2^ = 0.75) for Position 2 than for Position 1.

There were no significant effects of Sound Direction or interaction between Sound Direction and Position for any of the analysed parameters. We only observed a substantial though non-significant effect of Sound Direction for “time down” (F(2,48) = 2.41, *p* = 0.100, η^2^ = 0.09): participants took longer to return to the initial position with the ascending sound, though the effect did not reach significance (Constant: M = 2.85 s, SD = 0.94; Tone-up: M = 3.01 s, SD = 1.26; Tone-down: M = 2.94 s, SD = 1.16; see Supplementary Fig. S1).

#### Effects on task confidence

**Figure 2:**
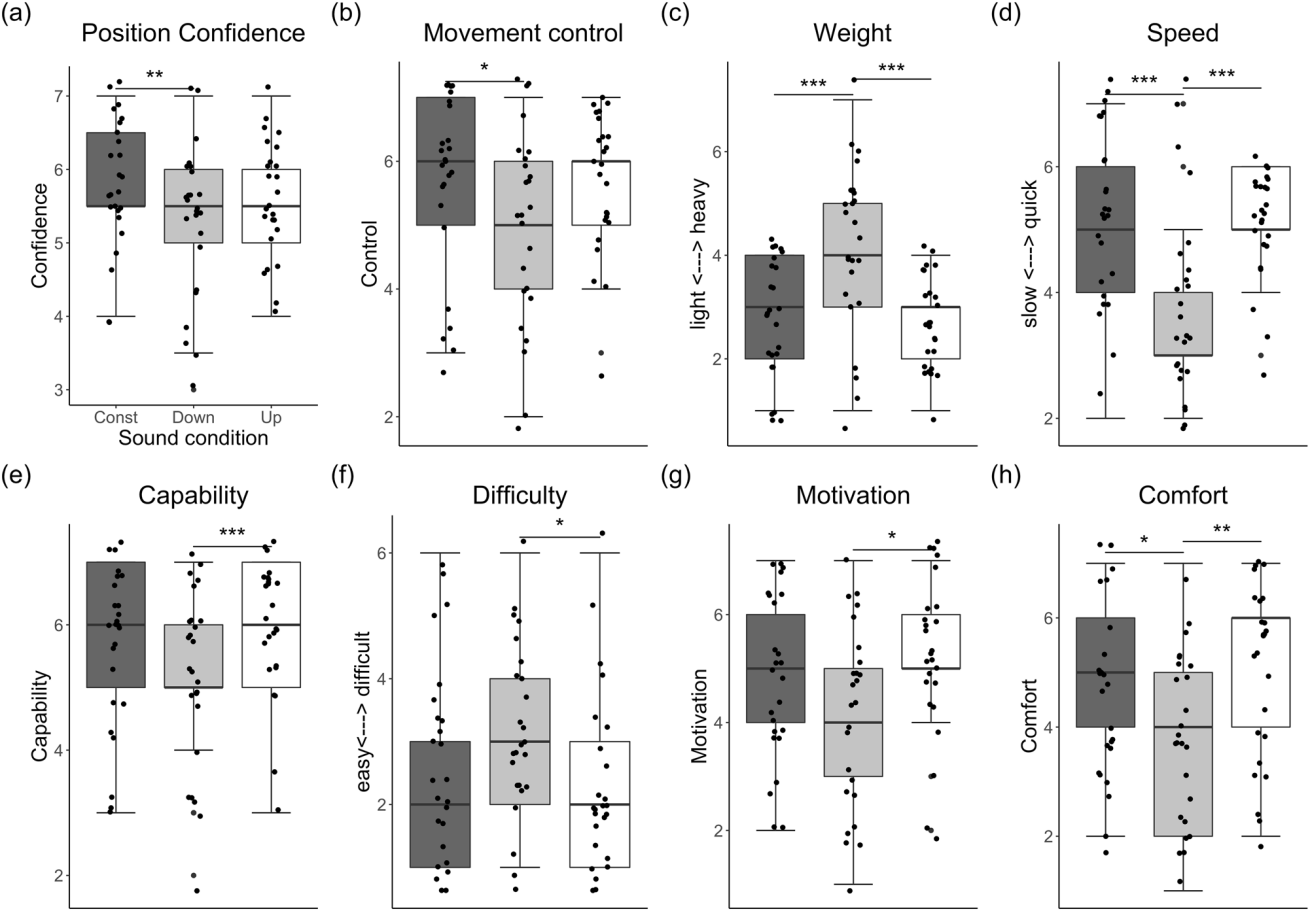
Boxplot with median (range) score for the feelings of confidence in having reached the requested position and feelings about one’s body and the bodily movement for all sound conditions in Experiment 1. (**a**) Feelings of position confidence (combining Position 1 y 2); (**b**) feelings of control over movement; (**c**) feelings of body weight and (**d**) speed; (**e**) felt capability and (**f**) difficulty to perform the exercise; (**g**) felt motivation and (**h**) comfort during the exercise. The asterisks indicate significant differences between sound conditions (* indicates *p* < 0.05, ** indicates *p* < 0.01, *** indicates *p* < 0.001; all corrected for multiple comparisons).

Sound Direction had an effect on Task Confidence (F(2,120) = 4.49, *p* = 0.037, η^2^ = 0.12), with no main effect of Position or interaction between factors. Participants were more certain about their hand position with the “Tone_constant” than with the “Tone_down” (t(120) = 2.98, *p* = 0.01); no significant differences were found between the “Tone_up” and the other conditions (see Fig. 2a). A significant correlation was found between the task confidence ratings and the actual performance (i.e. maximum angle) for the “Tone_down” and Position 1 (r(25) = 0.53, *p* = 0.007): participants were more certain about their arm being at Position 1 when the maximum angle was higher, even though their accuracy was not improved (see correlation Fig. S2).

#### Effects on bodily and emotional feelings

Sound Direction had an effect on the sense of Control (F(2,48) = 3.54, *p* = 0.037, η^2^ = 0.13), with participants reporting having a larger sense of Control over their movement with “Tone_constant” than with “Tone_down” (t(48) = 2.47, *p* = 0.045), see Fig. 2b. There were no significant differences in Agency over the heard sounds across conditions (*p* > 0.97): for all conditions participants overall agreed that the sounds they heard were produced by them (see Table S1).

Sound Direction also had an effect on the sense of Lightness (F(2,48) = 15.29, *p* < 0.001, η^2^ = 0.38) and Speed (F(2,48) = 10.50, *p* < 0.001, η^2^ = 0.30). People felt lighter with the “Tone_up” (t(48) = -4.87, *p* < 0.001) and “Tone_constant” (t(48) = -4.70, *p* < 0.001) than with the “Tone_down” sound, as shown in Fig. 2c. They also felt faster with the “Tone_up” (t(48) = -3.91, *p* < 0.001) and “Tone_constant” (t(48) = 4.02, *p* < 0.001) than with the “Tone_down” sound (Fig. 2d).

Further, participants felt more capable of completing the exercise (F(2,48) = 7.41, *p* = 0.002, η^2^ = 0.23) with the “Tone_up” than with the “Tone_down” sound (t(48) = 3.85, *p* = 0.001) (Fig. 2e). They reported that the exercise was easier (F(2,48) = 3.83, *p* = 0.029, η^2^ = 0.13 ), with the “Tone_up” than with the “Tone_down” sound (t(48) = 13.54, *p* = 0.025; Fig. 2f) and feeling more motivated to perform the exercise (F(2,48) = 4.62, *p* = 0.015, η^2^ = 0.16), with the “Tone_up” than with the “Tone_down” sound (t(48) = 3.03, *p* = 0.011, Fig. 2g). Lastly, participant felt more in comfort (F(2,48) = 6.25, *p* = 0.004, η^2^ = 0.21) while performing the exercise with “Tone_up” than “Tone_down” (t(48) = 3.37, *p* = 0.004) and with “Tone_constant” than “Tone_down” (t(48) = 2.61, *p* = 0.032; Fig. 2h).

### Experiment 2: effects of harmonic content (sound direction and timbre)

#### Effects on bodily movement

The analyses of the movement data showed a significant effect of the factor Position for all parameters. As expected, for the condition where participants were asked to reach Position 2, participants reached a higher “peak angle” (F(1,24) = 417.69, *p* < 0.001, η^2^ = 0.79) and showed also a higher “mean angle” (F(1,24) = 346.56, *p* < 0.001, η^2^ = 0.54). This effect of Position was reflected also in longer “time up” (F(1,24) = 25.32, *p* < 0.001, η^2^ = 0.05) and “time down” (F(1,24) = 44.61, *p* < 0.001, η^2^ = 0.03), higher “velocity up” F(1,24) = 165.10, *p* < 0.001, η^2^ = 0.36) and “velocity down” F(1,24) = 48.52.47, *p* < 0.001, η^2^ = 0.23), and higher “acceleration up” F(1,24) = 187.47, *p* < 0.001, η^2^ = 0.17), for Position 2 as compared to Position 1.

Apart from this main effect of Position, we found additional effects related to sound condition for the parameters “peak angle”, “acceleration up” and “velocity up”. In particular, for “peak angle” we found a significant interaction between Sound Direction and Position (F(1,24) = 5.76, *p* = 0.024, η^2^ = 0.19), but post-hoc paired comparisons between Ascending and Descending sounds in the Position 1 and in the Position 2 were both not significant (both ps > 0.75). For “acceleration up” we found a significant interaction between Sound Direction and Position (F(1,24) = 6.25, *p* = 0.019, η^2^ = 0.21), a non-significant, but substantial, interaction between Sound Direction and Timbre (F(1,24) = 3.87, *p* = 0.06, η^2^ = 0.14) and a significant triple interaction between all factors (F(1,24) = 6.19, *p* = 0.020, η^2^ = 0.20). The interactions were follow-up by conducting separate ANOVAs for Position 1 and 2 with factors Sound Direction and Timbre. The ANOVA for Position 1 showed an effect of Sound Direction in upward acceleration (F(1,24) = 6.44, *p* = 0.02, η^2^ = 0.21), with higher acceleration for the Ascending (Normalized acceleration: M = 0.127, SD = 0.049) than for the Descending sound (M = 0.121, SD = 0.048; see Supplementary Fig. S3a), and no significant effect of Timbre or interaction between factors (*p* = 0.6). For Position 2, there was only a significant interaction between Sound Direction and Timbre (F(1,24) = 8.08, *p* = 0.009, η^2^ = 0.25); but the follow-up paired t-test comparisons were all non-significant.

For “velocity up”, we found a main effect of Timbre (F(1,24) = 6.30, *p* = 0.019, η^2^ = 0.001), as participants were faster raising the arm when listening to the “Tone” sounds (M = 36.13, SD = 12.07 degrees/s) than the “Musical” sounds (M = 35.51, SD = 12.11 degrees/s). Moreover, for “velocity up” there was a triple interaction of the factors Sound Direction, Timbre, and Position (F(1,24) = 5.96, *p* = 0.022, η^2^ = 0.20). Follow-up separate ANOVAs for Position 1 and 2 with factors Sound Direction and Timbre showed that for Position 1 there was a significant effect of Timbre (F(1,24) = 5.89, *p* = 0.023, η^2^ = 0.20) and an interaction between Sound Direction and Timbre (F(1,24) = 4.61, *p* = 0.041, η^2^ = 0.16; see Supplementary Fig. S3b); paired t-test comparisons were all non-significant. For Position 2 there were no significant effects or interactions (F(1,24) = 0.58, *p* = 0.45, η^2^ = 0.02).

#### Effects on task confidence

**Figure 3:**
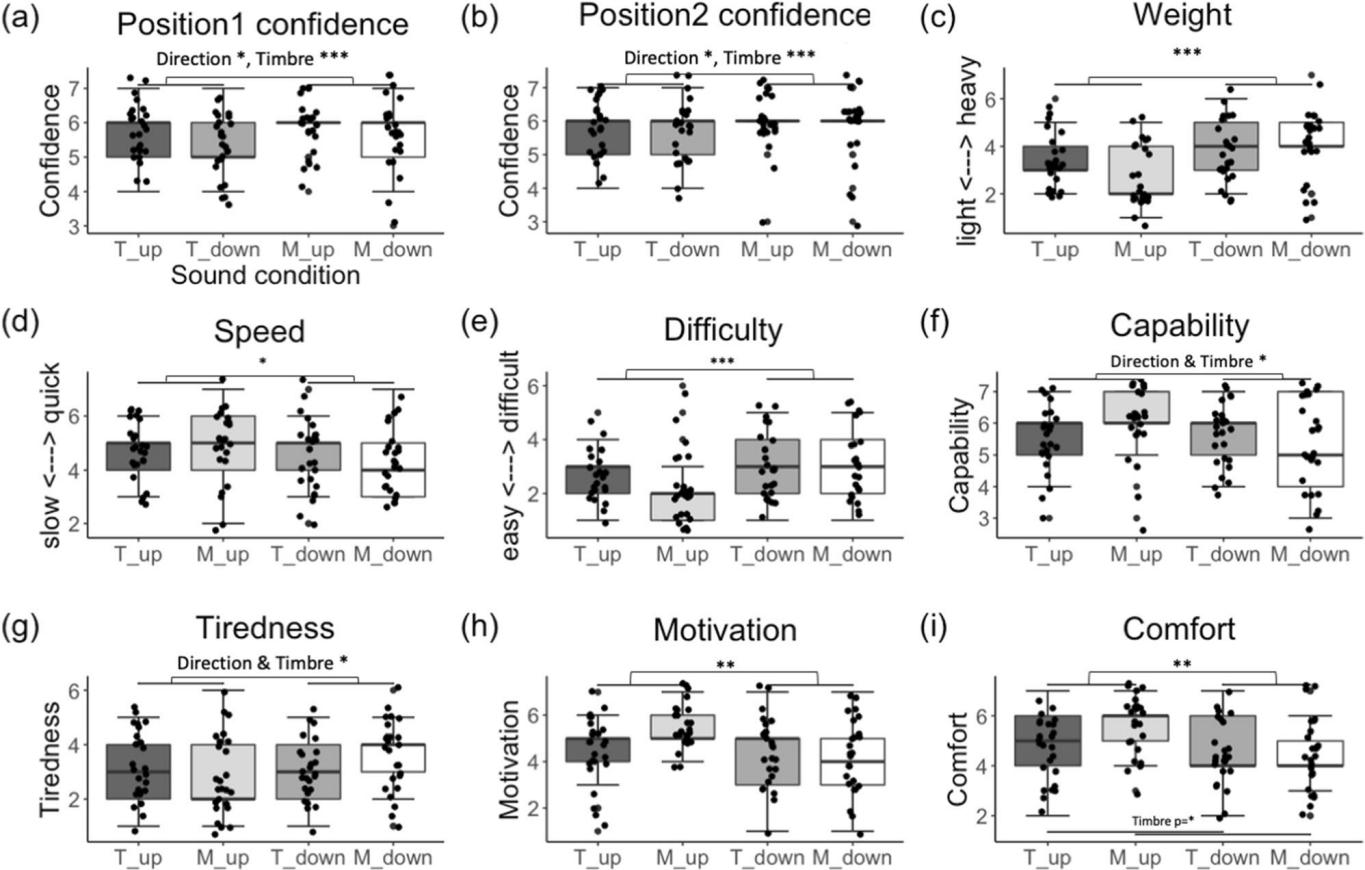
Boxplot with median (range) score for the feelings of confidence in having reached the requested position, feelings about one’s body and the bodily movement for all sound conditions in Experiment 2. (**a**) Feelings of position confidence in Position 1 and (**b**) Position 2; (**c**) feelings of body weight and (**d**) speed; (**e**) feelings of difficulty, (**f**) capability, (**g**) tiredness, (**h**) motivation and (**i**) comfort during the exercise. T_up = “Tone_up”, T_down = “Tone_down”, M_up = “Musical_up”, M_down = “Musical_down”. Note that in (**a**) and (**b**), related to position confidence, for the conditions Musical_up in both positions and Musical_down in Position 2 we observe a large concentration of participants’ answers around point 6 of the scale, suggesting that participants felt quite confident about their position with only few participants deviating from point 6. For the other conditions, we observe left- or right-skewed data, due to a larger dispersion in participants' responses. The asterisks indicate significant differences between sound conditions (* indicates *p* < 0.05, ** indicates *p* < 0.01, *** indicates *p* < 0.001; all corrected for multiple comparisons).

Task Confidence was significantly affected by the factors Sound Direction (F(1,71) = 4.83, *p* = 0.031, η^2^ = 0.063), Timbre (F(1,71) = 10.51, *p* = 0.001, η^2^ = 0.12), and Position (F(1,97) = 7.96, *p* = 0.005, η^2^ = 0.075), with no significant interaction between factors (all *p* > 0.062). As shown in Fig. 3a and b participants were more certain about their hand position with the Ascending than with the Descending sounds, with the “Musical” than with the “Tone” sounds, and in Position 2 than in Position 1.

A significant correlation was found between the task confidence ratings and the actual performance (i.e. maximum angle) for the “Tone_up” (r(25) = 0.40, *p* = 0.047), “Musical_up” (r(25) = 0.49, *p* = 0.013) and “Musical_down” (r(25) = 0.44, *p* = 0.028) conditions. Follow-up separate correlation analyses for Position 1 and 2 revealed significant correlations only for the “Musical_up” sound, both in Position 1 (r(25) = 0.45, *p* = 0.024) and Position 2 (r(25) = 0.50, *p* = 0.01). As in Experiment 1, for all conditions participants were more certain about their arm being at the requested position when the maximum angle was higher, even though their accuracy was not improved (see correlation Fig. S2).

### Effects on bodily and emotional feelings

In terms of Control over their movement and Agency over the heard sounds, there were no significant differences between conditions (all ps > 0.19). Participants agreed that they felt in control of their movements and that the sound was produced by them in all conditions (see Supplementary Table S2).

Sound Direction had a significant effect in feelings of Lightness, Speed, Difficulty, Motivation and Comfort: as shown in Fig. 3, participants felt lighter (F(1,71) = 12.69, *p* < 0.001, η^2^ = 0.15)), faster (F(1,71) = 5.39, *p* = 0.023, η^2^ = 0.070)), and reported that the exercise was easier (F(1,71) = 13, *p* < 0.001, η^2^ = 0.15), that they were more motivated to complete it (F(1,71) = 9.21, *p* = 0.003. η^2^ = 0.11) and felt more in comfort (F(1,71) = 9.89, *p* = 0.002, η^2^ = 0.12) with the ascending sounds (Up conditions) as compared to the descending sounds (Down conditions).

Moreover, Sound Timbre showed a main effect for feelings of Comfort (F(1, 71) = 4.65, *p* = 0.034, η^2^ = 0.06), with participants reporting a higher feeling of comfort in the “Musical” sounds than in “Tone” sounds, as it can be seen in Fig. 3.

Finally, we observed a considerable, though not significant, interaction effect between the factors Sound Direction and Timbre for the feelings of Lightness (F(1,71) = 3.57, *p* = 0.062, η^2^ = 0.04) and exercise Difficulty (F(1,71) = 3.59, *p* = 0.061, η^2^ = 0.048). This interaction reached significance for the feelings of Capability (F(1,71) = 4.93, *p* = 0.029, η^2^ = 0.06), Tiredness (F(1,71) = 5.49, *p* = 0.021, η^2^ = 0.071), Motivation F(1,71) = 5.99, *p* = 0.016, η^2^ = 0.077) and Comfort (F(1,71) = 3.87, *p* = 0.052, η^2^ = 0.051). The interactions were driven by the fact that the difference between Ascending and Descending sounds was larger for the Musical than for the Tone sounds: to a larger extent in the case of the Musical sounds, with ascending vs descending sounds participants felt lighter (X^2^(1) = 3.66, *p* = 0.056), more capable (X^2^(1) = 5.18, *p* = 0.023), less tired (X^2^(1) = 5.54, *p* = 0.018), more motivated (X^2^(1) = 6.10, *p* = 0.014), more comfortable (X^2^(1) = 4.05, *p* = 0.044), and found the exercise easier (X^2^(1) = 3.81, *p* = 0.05) (Fig. 3).

### Experiment 3: effects of absolute frequency range (sound direction and frequency range)

#### Effects on behavior (bodily movement)

The analyses of the movement data showed a significant effect of the factor Sound Frequency Range on Peak Angle (F(1,19) = 5.71, *p* < 0.027, η^2^ = 0.23). The results showed that a higher peak angle was reached with “High Pitch” (M = 101.32, SD = 13.70 degrees) than with “Low Pitch” sounds (M = 100.06, SD = 11.89 degrees; see Supplementary Fig. S4). Since the requested position was 90 degrees, participants were more accurate in their reached position for the “Low Pitch” sounds.

#### Effects on bodily feelings

**Figure 4:**
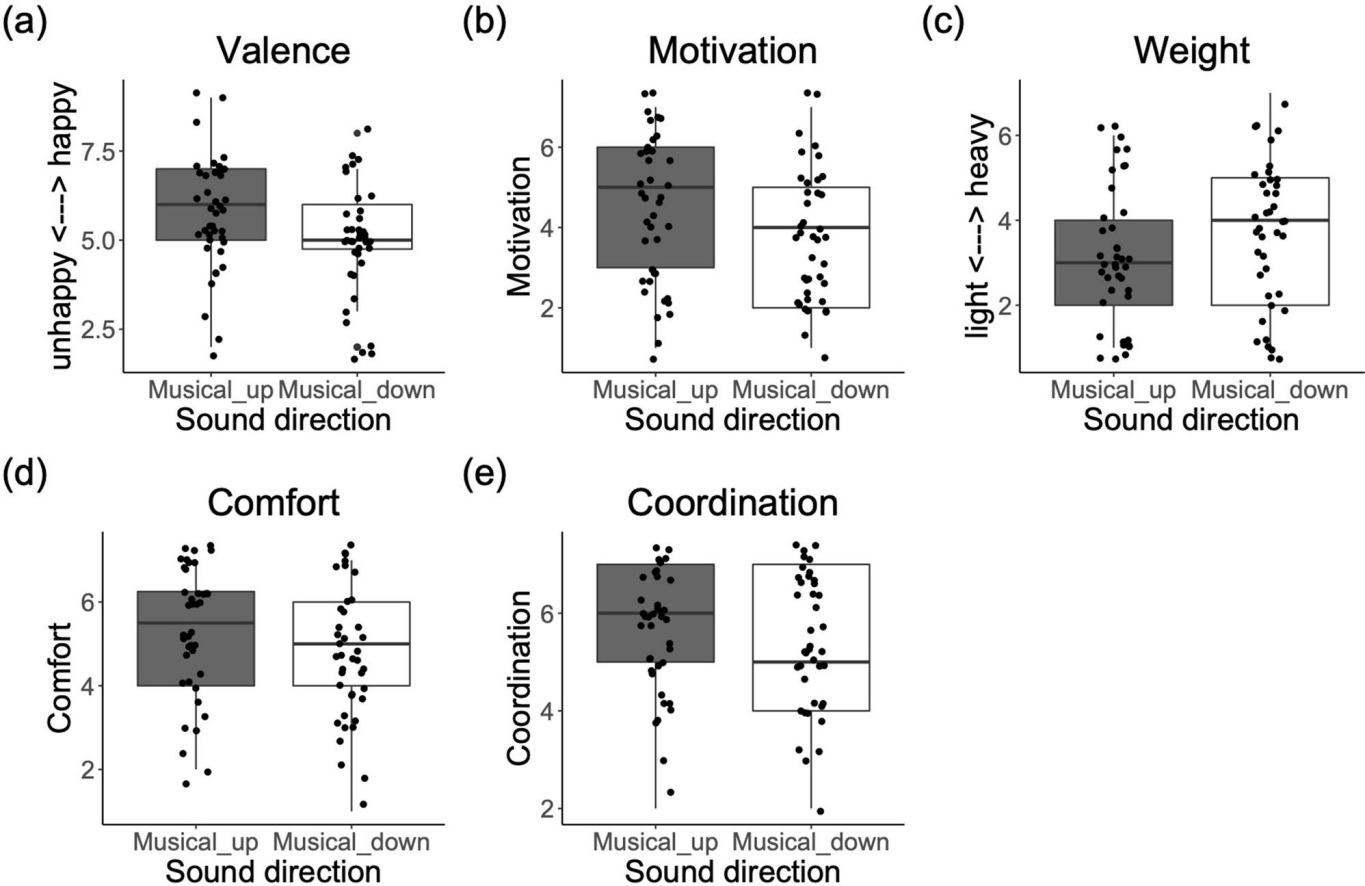
Boxplots with median (range) score for questionnaire items showing significant effects of Sound Direction in Experiment 3. (**a**) Reported emotional valence (happiness) and (**b**) motivation; and (**c**) feelings of body weight, (**d**) movement comfort and (**e**) coordination.

Sound Direction had a significant effect on feelings of body Weight, Comfort and Coordination: participants felt lighter (F(1,54) = 4.42, *p* = 0.040, η^2^ = 0.075), more comfortable (F(1,54) = 6.42, *p* < 0.014, η^2^ = 0.106) and with more coordinated movements (F(1,54) = 4.35, *p* = 0.041, η^2^ = 0.074), with the "Musical_up” than with the “Musical_down” sounds (Fig. 4). Furthermore, we observed a substantial, although not significant, effect, of Sound Direction on Speed (*p* = 0.060), as participants felt considerably faster with the “Musical_up” than with the “Musical_down” sounds (see Supplementary Table S3).

**Figure 5:**
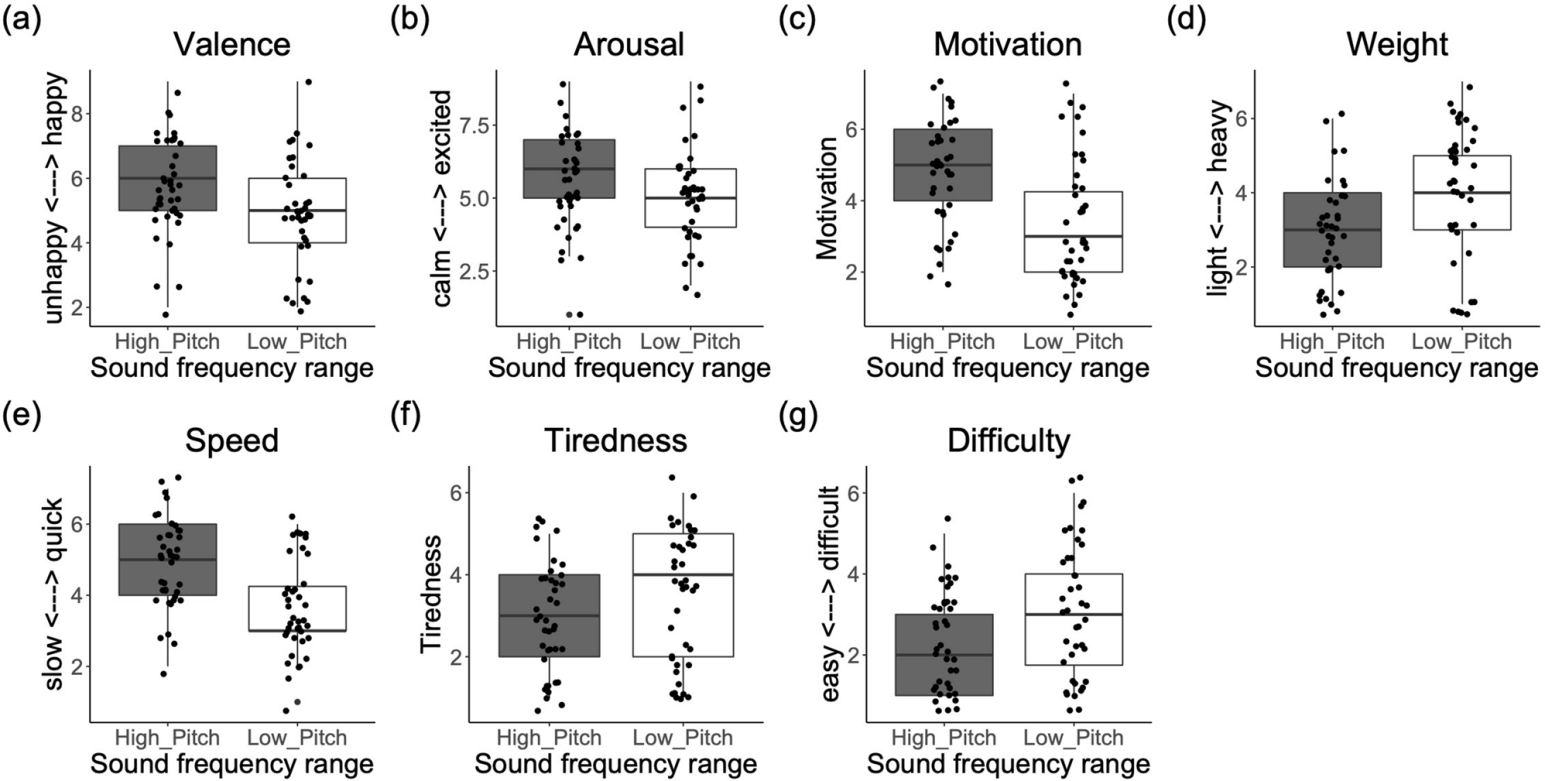
Boxplots with median (range) score for questionnaire items showing significant effects of Sound Frequency Range in Experiment 3*.* (**a**) Emotional valence (happiness), (**b**) arousal (excitation) and (**c**) motivation; (**d**) feelings of body weight, (**e**) speed, and (**f**) tiredness; and (**g**) movement difficulty.

With regards to Sound Frequency Range, there were effects on feelings of body Weight, Speed, Tiredness and Difficulty. “High pitch” caused participants to feel lighter (F(1,54) = 21.07, *p* = 0.001, η^2^ = 0.281) and faster (F(1,54) = 28.31,* p* = 0.001, η^2^ = 0.34) than “Low_pitch” sounds. Further, the “Low_pitch” made participants feel more tired (F(1,54) = 13.10, *p* = 0.001, η^2^ = 0.195) and with more difficulty (F(1,54) = 6.37, *p* = 0.014, η^2^ = 0.105) to perform the exercise than the “High_pitch” sound, see Fig. 5.

There was a significant interaction between Sound Frequency Range and Direction in relation to feelings of Tiredness (F(1,54) = 7.05, *p* = 0.010, η^2^ = 0.115). Follow-up tests showed that the “Musical_down_Low_pitch” sound caused participants to feel significantly more tired than the other sound conditions (X^2^(1) = 5.54, *p* = 0.018) (see Supplementary Table S3 and Fig. S5). There was also a substantial, though not significant, interaction effect between Sound Direction and Sound Frequency Range for Coordination (F(1,54) = 3.84, *p* = 0.066, η^2^ = 0.061), which was mainly due to the “Musical_down_Low_pitch” sound causing participants to feel less coordinated than the other sound conditions (see Supplementary Table S3 and Fig. S5).

Finally, in terms of Strength, Capability, movement Control, and Agency over the heard sounds, there were no significant effects (all ps > 0.12). For all conditions participants agreed that they felt in control and capable of their movements.

#### Effects on emotional feelings

Sound Direction had a significant effect on reported emotional Valence and Motivation. Participants felt happier (F(1,54) = 5.60, *p* < 0.021, η^2^ = 0.093) and more motivated (F(1,54) = 5.40, *p* = 0.020, η^2^ = 0.095) with the "Musical_up” than with the “Musical_down” sounds, independently of the Sound Frequency Range (see Fig. 4). The effect of Sound Direction on reported Arousal was not significant (*p* = 0.077), although results showed that participants felt considerably more excited with the “Musical_up” than with the “Musical_down” sounds (see Supplementary Table S3 with the median scores for all questionnaire items in relation to Sound Frequency Range and Sound Direction).

With regards to Sound Frequency Range, there was a significant effect on emotional Valence and Motivation, and a considerable, though non-significant effect, on Arousal. “High pitch” caused participants to feel happier (valence; F(1,54) = 4.14, *p* = 0.047, η^2^ = 0.071), more motivated (F(1,54) = 19.55, *p* = 0.001, η^2^ = 0.265) and more excited (arousal; F(1,54) = 3.12, *p* = 0.082, η^2^ = 0.054) than “Low_pitch” sounds (Fig. 5).

## Discussion

In this study we investigated how body-representation and feelings would be influenced by various sound characteristics when performing a basic movement that is accompanied by a sound. We looked more specifically at how bodily movement, proprioceptive awareness (sustained by body schema) and subjective feelings related to the body, one’s movement and emotional state, are affected by auditory changes. Across three experiments we studied the effects of pitch direction in tones (Experiment 1), richer musical features (Experiment 2) and absolute frequency range (Experiment 3). Overall, as shown in the summary Table 3 (see also Supplementary Table S4), changes in pitch had effects on the emotional state and on the various bodily dimensions investigated—bodily movement, proprioceptive awareness, bodily feelings—with more comprehensive effects observed for the latter explicit measures. Richer sound timbre and accentuated attack affected the confidence of participants about their body position, as well as the velocity of their movement, but mostly amplified the measured differences between ascending and descending pitch sounds in bodily feelings. Shifting the absolute frequencies of the descending pitch sounds impacted the amplitude of the performed movement (and therefore the accuracy of the final reached position according to the experiment task), and interacted with participants' feelings of tiredness. It also had a general effect on feelings of happiness and other bodily feelings. We discuss the mechanisms behind the observed effects and potential limitations, future work, and applications in the following subsections.

### Pitch change and bodily movement

Based on previous evidence (see Table 1), we had hypothesised that pairing movement with pitch changes, generally not associated with body movement, could be sufficient to lead to proprioceptive changes, as well as changes in movement and feelings, due to cross-modal correspondences between pitch change and motion on the vertical axis^29–31^. As suggested by previous works^13^ we hypothesize that the changes in perceived sound localization induced by sounds changing in pitch^66^ interact with internal models of body-representation. This suggestion is supported by neuroscientific studies showing an overlap between the brain areas that are activated by sounds rising/falling in pitch^67^, and the multisensory parietal areas integrating somatosensory, visual and auditory signals to form body-representations^68^. Areas activated sounds rising/falling in pitch include the right intraparietal sulcus, also activated by spatial words (such as “left” or “up”), and audio-visual motion areas (hMT +/V5 +)^67^. Our hypotheses are partly confirmed by our results. As shown in Table 3, the self-reports (with partial behavioural support) did reveal that the multisensory interaction between pitch and bodily movement can impact bodily experiences, and point towards a potential interference of sound with proprioception.

We found that a sound increasing or decreasing in pitch triggered at the beginning of the participant’s arm movement led participants to become less confident about their arm position than with a constant sound. Further, we found that, for the conditions with sounds changing in pitch, increases in participants confidence were not accompanied by increases in actual performance (i.e., more accurate position reached) but rather by overall increases in the reached angle, thus suggesting a degradation in the ability to judge accurately the arm position. Two recent studies have similarly suggested a potential interference of sounds changing in pitch with proprioception of a body part (one’s fingertip). The first study^13^ reported an “auditory Pinocchio”' illusion, in which participants feel and estimate their finger to be longer, elicited by presenting brief sounds rising in pitch with the action of oneself pulling on one’s occluded fingertip. This illusion was then replicated^37^ both in adults and pre-school children, when the experimenter, rather than the participants themselves, pulled the finger of the participant, thus suggesting that the effects of sound on the proprioception of one’s fingertip can take place both under passive and active touch conditions. Nevertheless, before the present study, it was unclear whether the effect of sounds changing in pitch on proprioception requires “anchoring” those sounds through tactile cues, as in the case of the “auditory Pinocchio”' illusion. In the classical version of the Pinocchio illusion, touch is also involved: participants touch their nose while their bicep tendon receives vibration, which causes the illusory feeling of one’s arm extending and, in turn, the illusion of elongation of one’s represented nose (^69^; see related studies^70,71^). Other studies involving vision also use touch as an “anchor” (e.g., in the rubber-hand illusion^7^; see related out-of-body illusion^16^) or may not need this “anchor” as they involve vision of body parts, for instance, live video feed showing an altered position of one’s hands that leads participants to mislocalize the position of their hands (^72,73^; see also^56^). Our study thus shows that sounds can affect body representations in the absence of tactile anchor: changes in pitch height interfere with internal proprioceptive signals and alter the perceived position of limbs if paired and presented synchronously with body movement and with no other sensory signals involved (such as vision or touch).

Other studies with vision showed that altering the perceived feedback on the position of a moving limb results in adjustment in the participant’s limb movement^56^. Our study shows similar effects of changing pitch on bodily movement. The arm movement amplitude was not strongly affected, but in Experiment 2 the movement acceleration triggered by the ascending pitch sound was. We hypothesize that this increase in acceleration probably reflects a compensatory response mediated by auditory feedback signaling a discrepancy between the predicted position and the received sensory feedback. Such corrections in velocity/acceleration have been observed in other studies providing altered sound feedback, for instance, on the trajectory of a movement^74^ or on the applied weight on the floor when walking^75^. They may relate to the forward model theories for motor control^76^, in which large discrepancies between the predicted and actual sensory inputs generate such compensatory patterns. Such corrections are actually beneficial and sought in some contexts, such as sports or physical rehabilitation, as part of sensory-motor entrainment (^77^; for recent reviews see^78,79^).

Further, we found that sounds changing in pitch affect participants' feelings about their body and their movement. With the ascending sound participants feel, for instance, lighter and quicker, and find the upwards movement easier to perform, than with the descending sound. We attribute this feeling to the perception of being “pushed up” by the ascending sound, which is compatible with the facilitation of the upwards movement. We also found an effect on emotional state, in line with the affective correspondence between raising pitch and happiness^20,21^.

Future research should investigate whether these effects of the ascending vs. descending sound reverse or hold for a downwards movement, as previous studies on the “auditory Pinocchio” illusion showed that the effect does not reverse when inverting the direction of finger pulling^13^.

### Harmonic content and bodily movement

Experiment 2 shows that musical sounds, as compared to the tone sounds, made people feel more confident of having reached the requested position, increased the feeling of comfort in performing the movement and decreased the velocity of the upwards movement. While confidence increased with harmonic complexity, such increases in confidence were not accompanied by increases in actual performance.

As these effects occurred both for ascending and descending sounds, they may relate to the emotional processes triggered by the musical sounds, as these sounds are generally more pleasant to listen to than pure tones (see^80^ for low frequency). Musical sounds are also ecologically more valid: they are closer to sounds that the participants are familiar with, while the tone sounds are typically found only in specific electronic devices. They also provide more possibilities in terms of designing sonification that may impact body movement. Previous studies have shown that the mapping of musical features (e.g., tempo, pitch, etc.) to movement properties (e.g., movement velocity, body inclination) can be used to improve the understanding of full-body movement and expressive gestures and to drive movement behavior^81,82^. For instance, Newbold et al. showed that musical structures could be embedded into the sonification of movement to manipulate the feeling of wanting to continue or conclude a movement and the feeling of accomplishment^32,49^. Note that differently from those studies, here we used sounds that do not provide accurate spatial information about the movement: once the sound is triggered this sound does not change according to movement (i.e., we do not sonify movement) and the sound is irrelevant to the movement task. Our participants highly likely never experienced such coupling of their movement with sound; thus, we can assume they did not have any clear expectancy on the movement-sound coupling.

Beyond this general effect, results also showed that the musical features interacted with the overall effect of changes in pitch in several bodily feelings. The musical effect was not that of purely enhancing the bodily effects in the positive direction, but on increasing the difference between ascending and descending pitch sounds for the musical versus the pure tone sounds. People felt even lighter and more comfortable with the ascending vs. the descending musical sound, than they did for the pure tones. People felt less tired with the ascending musical sound and more tired with the descending musical sound, than they did for the pure tones. Feelings of capability and motivation to perform the movement were lower for the descending musical sound than for the other sounds, while the movement seemed easier with the ascending musical sound than for the other sounds. The effects on pitch—especially on subjective/direct measures—could be linked to the “semantic” explanation of the cross-modal pitch correspondences, where high pitch is supposed to be more active and low pitch more passive^30,83^ (see also^42^). In the same line, Apple et al.^84^ found that people judged other people with higher pitched voices as less potent.

In relation to this interaction effect, previous studies have shown that dynamic changes in loudness, such as those present in our musical sound interact with the perception of pitch^23–26,85^. Furthermore, changes in loudness on their own can already elicit an impression of movement changes^27,28^. Hence, these changes in loudness may be responsible for maximizing the crossmodal correspondences between pitch and upward/downward space. Nevertheless, as musical sounds with richer spectrum are also more pleasant to listen to than pure tones, as said above, the interaction effect may derive from the fact that crossmodal correspondences between pitch and upward/downward space are emotionally mediated, as previously suggested (^20^; see also^21,22^ for reviews). Our current data does not allow us to make any conclusive remarks on the origin of the interaction effects between the effect of pitch and the musicality of the sound, and this should be explored in future studies.

### Absolute frequency range and bodily movement

In Experiment 3, we found that shifting the absolute frequencies of the changing pitch sound impacted the movement, as well as the emotional state, and bodily feelings, of the participants. In particular, the higher frequency sound increased the amplitude of the participants’ arm raise movement, making the arm reach a higher peak angle. People felt happier, lighter, less tired, more motivated and found the movement easier with the higher frequency sound as compared to the lower frequency sound. Because these effects were independent of the movement direction, they probably relate to two different processes. On the one hand, we must consider that apart from vertical space, pitch is associated with physical size. High and low pitches are respectively congruent with smaller and larger sizes, as shown for the perception of object size^40–44^ and for the perception of people’s body size^86,87^. Shifting the pitch of one’s own bodily produced sounds (i.e., footstep sounds) has been shown to change how people perceive their own body size and weight^12^ and also to make people find exercises less difficult and feel less tired^33^. Similarly, in our current study the feelings of being lighter, less tired, more motivated and finding the exercise easier, which were elicited by the high pitch sounds, may have pushed participants to raise their arms higher. Second, the emotional processes triggered by the high frequency sounds, which made participants feel happier, as shown by the results, may have interacted with the bodily feelings, or both processes may have influenced each other. Our findings here relate to those from a previous qualitative study where bodily movement was paired with a sound rising in pitch similar to the musical sound we employed in Experiment 3; this study suggested that this sound could lead to more pleasantness and feelings of movement fluidity, body lightness and flexibility in the context of exertion^45^.

Beyond this general effect of pitch, results showed that the absolute frequency range interacted with the effect of the change in pitch (i.e., the relative pitch) in the feelings of tiredness. The interaction effect showed that the difference between ascending and descending pitch sounds was amplified for the low pitch versus the high pitch sounds. With the low pitch sounds people felt less tired with the ascending sound than with the descending sound, but with the high pitch the change in pitch did not seem to have an effect and people felt overall less tired than with the low pitch sounds. Several studies also show that the association of pitch variation and frequency ranges with movement does not follow simple rules and can be highly influenced by the context^88^. In^31^, by asking participants to describe sounds gesturally, they confirm that pitch variations are typically associated with spatial metaphors. While low pitch and high pitch are generally linked to low and high hand positions, some participants nevertheless used different associations, for example lateral displacements with pitch. This is found in several musical instruments such as the keyboard. The case of the cello is also noteworthy, since the high pitches are obtained by lowering the hand. Other concepts such as action of efforts could also explain the association of pitch variation with movements. In the context of Indian music, pitch glides or melodic contour can be associated with “physical effort”^89,90^. The relationship between effort, motion and sound has also been reported in the case of electric guitar performances in^91^. Finally, it should be noted that different asymmetries have been found whether sound parameters increase or decrease, even if absolute changes are identical. Pitch perception and its association to movement can depend on the direction^92^. Sounds with increasing intensity sounds are perceived as being longer and their range of loudness appears to be higher^93^. More investigations are necessary to disentangle the different possible cross-modal associations of sound features, also in relation with people skills and background^94^.

### Limitations and future research

Systems that provide sound feedback on movement in real-time have been found to increase bodily awareness and influence movement (e.g.,^46,47^) and are increasingly being used in the context of musical expression^95^, dance (e.g.,^96^), sports (e.g.,^97–99^), general physical activity (e.g.,^38,45^) and physical rehabilitation (e.g.,^100,101^) for example, in people with chronic stroke^102–105^, vestibular disorders (e.g.,^106,107^), chronic pain (e.g.,^47,49^) or autism (e.g.^108^). By contrast with those movement sonification scenarios, our studies do not rely on continuous real-time adjustment of the sound once it has been triggered. The interaction between the sound and the movement differs then from direct sonification in which body movement is tracked and mapped into real-time auditory feedback providing information on the movement itself. In this regard, the type of sound feedback chosen in our studies is closer to dance practices where movement adjustment occurs generally by anticipating to and synchronising with sound at key moments.

In the present study, we focused on ascending and descending pitch sounds because of their reported association with changes in motion along the vertical plane (i.e., upwards and downwards motion). However, only the upwards part of the movement was paired with sounds. To better study the congruence between pitch and motion direction, future work could focus on investigating the effect of ascending and descending pitch sounds accompanying both upwards and downwards movement. While in the present study the movement chosen (arm raise) demands effort only on the upwards part of the movement, a movement or exercise similarly demanding on both upwards and downwards movement may be better chosen for such investigations. Furthermore, future research may consider comparing the effects of the sounds changing in pitch to a “no sound” condition; note that in the present study the constant sound was included in Experiment 1 as a “control” or reference condition with which to compare the effect of the sounds changing in pitch (as in^13,37^), as opposed to a “no sound” condition, in order to control for the effect of simply listening to a sound.

Extending the research to other bodily movements is certainly important in order to generalise the findings. Still the arm raise exercise chosen for this study is a generic gesture that is often part of dance sequences and of many warm-up or toning routines of programs oriented to dance, general physical activity (e.g.,^54^) or physical rehabilitation (e.g.,^109^). In a previous qualitative study^45^, participants were asked to use the ascending pitch musical sound paired with bodily movement in the context of exertion, and they used it to accompany squat and step-up exercises. For these exercises, there were reports of changes in bodily feelings (movement fluidity, body lightness and flexibility) which suggests that our findings may generalise to those movements. Nevertheless, quantitative data is needed to better understand the effects. Including longer movement sequences may reveal different effects on bodily movement than the ones observed in this study.

In Experiment 2, we found an interaction effect between the harmonic content (i.e., timbre) and the overall effect of pitch in several bodily feelings. Since the aesthetic or affective aspects of sounds may change people’s overall emotional states and/or change the multisensory integration (e.g.,^110^), we cannot exclude that this interaction may derive from the fact that crossmodal correspondences between pitch and upward/downward space are emotionally mediated^20–22^. However, since Experiment 2 did not include a measure of emotional state (beyond motivation), our current data does not allow us to make any conclusive remarks in this respect. Future research should include emotional measures, both self-report and physiological real-time measures (as in^12^), to clarify the origin of the interaction. Similarly, future research should disentangle the possible interaction effects between emotional feelings and bodily feelings observed in Experiment 3, as well as the potential effects of individual differences (e.g., due to sound and music skills, dance or movement skills, etc.)^45^.

Finally, we should note that our explicit measures revealed more consistent effects than the implicit measures. While the measures employed to assess implicit body-representations did not reveal large effects, these may nonetheless come to affect explicit representations^111^. Dissociation between implicit and explicit body-representation measures is frequently observed in the literature^6,11,13,112^. For instance, preschool children, by contrast with adults, showed a so-called “auditory Pinocchio” illusion only when subjective feelings were considered, but this did not translate in their perceived finger position, suggesting that multisensory interactions contribute differently to subjective feelings and sense of position and depending on developmental stage^37^. Future research should also consider the inclusion of measures of interoception, as movement also goes in accordance with movements from inside the body. For instance, some works have shown that people move at the beat of their own heartbeat^113–115^. Hence, the body movement observed in these experiments (as a function of sound) might relate to both internal bodily modulations (e.g., from the cardiovascular system) and external bodily modulations (e.g., music and other cues present in the general environment).

From an application point of view, while there might be value in understanding these effects to have specific influences in fine movements, such as in dance or sports contexts, our findings may be most useful to understand how music and sound triggers changes in perceived body capabilities and positive feelings about one’s body. In this regard, our work contributes to the human–computer interaction and sonification research that focuses on inviting movement and helping overcoming psychological barriers related for instance to fear of injury or lack of confidence in one’s movement, for instance, in people who are physically inactive^33,38,45,52^ or in rehabilitation of conditions such as chronic pain^46–49^. Embedding these psychological factors related to body perception into the design process of applications to support dance or physical activity opens opportunities for movement expression and clinical applications.

## Supplementary Information

Supplementary Information 1.

Supplementary Information 2.

Supplementary Information 3.

Supplementary Information 4.

Supplementary Information 5.

Supplementary Information 6.

Supplementary Information 7.

## Data Availability

All data generated or analyzed during the experiments is available in the Supplementary Material.
