# Supplementary material for: Effects of pitch and musical sounds on body-representations when moving with sound
Source: Sci Rep. 2022 Feb 17;12:2676. doi: 10.1038/s41598-022-06210-x (PMC8854572; doi:10.1038/s41598-022-06210-x)
Supplement: Supplementary file 7 — Supplementary Information 7. [file 41598_2022_6210_MOESM7_ESM.pdf]

**Bodies moving with sound: Effects of pitch and musical sounds on body-representations**

Judith Ley, Eslam Alshami, Aneesha Singh, Frédéric Bevilacqua, Nadia Bianchi-Berthouze,

Ophelia Deroy and Ana Tajadura-Jiménez

**Table S1**. Median (range) for questionnaire items (7-point Likert-type items) for the three sound conditions in Experiment 1. * marks significant median differences between conditions.

| **Variables** | | **Tone** | | |
|---|---|---|---|---|
| | | **Up** | **Down** | **Constant** |
| Confidence* | Position 1 | 6 (4-7) | 5 (3-7) | 6 (3-7) |
| | Position 2 | 6 (3-7) | 6 (3-7) | 6 (3-7) |
| Body weight* | | 3 (1-7) | 4 (1-4) | 3 (1-5) |
| Control* | | 6 (2-7) | 5 (3-7) | 6 (3-7) |
| Speed* | | 5 (2-7) | 3 (3-6) | 5 (2-7) |
| Tiredness | | 3 (1-6) | 3 (1-6) | 3 (1-5) |
| Difficulty* | | 2 (1-6) | 3 (1-6) | 2 (1-6) |
| Capability* | | 6 (2-7) | 5 (3-7) | 6 (3-7) |
| Agency | | 5 (1-7) | 5 (2-7) | 5 (2-7) |
| Motivation* | | 5 (1-7) | 4 (2-7) | 5 (2-7) |
| Comfort* | | 6 (1-7) | 4 (2-7) | 5 (2-7) |

**Table S2.** Median (range) for questionnaire items (7-point Likert-type items) for all sound conditions in Experiment 2. *D, *T, *P and D*T mark significant median differences due to, respectively, Sound Direction, Timbre, Position and interaction between Sound Direction and Timbre. ^ marks trends towards significance.

| Variables | | Tone | | Musical | |
|---|---|---|---|---|---|
| | | Up | Down | Up | Down |
| Confidence *D, *P, *T | Position 1 | 6 (4-7) | 5 (4-7) | 6 (4-7) | 6 (3-7) |
| | Position 2 | 6 (4-7) | 6 (4-7) | 6 (3-7) | 6 (3-7) |
| Body weight *D D^T | | 3 (2-6) | 4 (2-6) | 2 (1-5) | 4 (1-7) |
| Control | | 6 (3-7) | 6 (2-7) | 6 (2-7) | 6 (1-7) |
| Speed *D | | 5 (3-6) | 5 (2-7) | 5 (2-7) | 4 (3-7) |
| Tiredness D*T | | 3 (1-5) | 3 (1-5) | 2 (1-6) | 4 (1-6) |
| Difficulty *D D^T | | 3 (1-5) | 3 (1-5) | 2 (1-6) | 3 (1-5) |
| Capability D*T | | 6 (3-7) | 6 (4-7) | 6 (3-7) | 5 (3-7) |
| Agency | | 5 (1-7) | 5 (1-7) | 6 (1-7) | 5 (1-7) |
| Motivation *D D*T | | 5 (1-7) | 5 (1-7) | 5 (4-7) | 4 (1-7) |
| Comfort *D *T D*T | | 5 (2-7) | 4 (2-7) | 6 (3-7) | 4 (2-7) |

**Table S3.** Median and range questionnaire items (7-point Likert-type items, except for valence and arousal which were 9-point Likert-type) for all sound conditions in Experiment 3. *D, *F and D*F mark significant median differences due to, respectively, Sound Direction, Frequency range and interaction between Sound Direction and Frequency Range. ^ marks trends towards significance.

| Variables | | Musical Up | | Musical Down | |
|---|---|---|---|---|---|
| | | High | Low | High | Low |
| 7-point Likert | Body Weight *D *F | 3 (1-6) | 4 (1-6) | 3 (1-6) | 5 (1-7) |
| | Control | 6 (2-7) | 6 (2-7) | 6 (2-7) | 5 (1-7) |
| | Speed ^D | 5 (2-7) | 4 (2-6) | 4.5 (3-7) | 3 (1-6) |
| | Tiredness *F D*F | 3 (1-5) | 3.5 (1-6) | 3 (1-5) | 4.5 (1-6) |
| | Difficulty *F | 2 (1-4) | 3 (1-6) | 2.5 (1-5) | 3.5 (1-6) |
| | Capability | 6 (2-7) | 6 (2-7) | 5 (1-7) | 5 (2-7) |
| | Agency | 2.5 (1-6) | 3 (1-7) | 3 (1-6) | 2.5 (1-5) |
| | Motivation *D *F | 5 (2-7) | 4 (1-7) | 5 (2-7) | 3 (1-6) |
| | Comfort *D | 5.5 (2-7) | 5.5 (2-7) | 5 (1-7) | 5 (2-7) |
| | Strength | 5 (2-7) | 5 (2-7) | 4.5 (1-7) | 4 (2-7) |
| | Coordination D* D^F | 5.5 (3-7) | 6 (2-7) | 6 (3-7) | 5 (2-7) |
| 9-point Likert | Valence *D *F | 6 (3-9) | 5 (2-9) | 5 (2-8) | 5 (2-7) |
| | Arousal ^D *F | 6 (1-9) | 5 (2-9) | 5 (3-8) | 5 (2-8) |

**Table S4.** Detailed summary of results on the effects across all the experiments.

| Dimension | | Predicted effects | Pitch change | Added Timbre (vs Tone) | Absolute frequency range |
|---|---|---|---|---|---|
| Bodily movement | | Amplitude | Not affected | Not affected | Increase in movement amplitude (i.e. a higher peak angle) for the “High Pitch” vs “Low Pitch” sounds |
| | | Acceleration/ velocity | Higher maximum upwards acceleration for ascending vs descending pitch | Higher velocity on the upwards movement for the “tone” vs “musical” sounds | Not affected |
| Proprioceptive awareness | | Accuracy of final position | Not affected | Not affected | More accurate for the “Low Pitch” sounds |
| | | Confidence on perceived final position | Feeling more uncertain about their hand position with the descending pitch vs the constant sound (Exp. 1) and vs the ascending pitch sound (Exp. 2) | “Musical” sounds made participants feel more confident about the reached position than “Tone” sounds | Not assessed |
| | | Sense of control | Larger sense of control over the movement with constant vs descending sound | Not affected | Not affected |
| Bodily feelings and emotional state | Feelings about the body | Weight | Feeling heavier with the descending vs. the ascending or constant sound | The “Musical” sounds maximize the differences between ascending and descending sounds | Feeling lighter with the “High” vs “Low” pitch |
| | | Speed | Feeling slower with the descending vs. the ascending or constant sound | Not affected | Feeling faster with the “High” vs “Low” pitch |
| | | Tiredness | Feeling more tired with the descending vs. the ascending sound | The “Musical” sounds maximize the differences between ascending and descending sounds | Feeling less tired with the “High” vs “Low” pitch<br><br>Interaction between Sound Direction and Frequency Range: The “Musical_down_Low_pitch” sound made participants feel more tired |

| | | | | | |
|---|---|---|---|---|---|
| | | Strength | Not affected | Not assessed | Not affected |
| | Feelings about the movement | Sense of control | Larger sense of control over the movement with constant vs descending sound | Not affected | Not affected |
| | | Ease | Exercise felt easier with the ascending vs descending sound | The “Musical” sounds maximize the differences between ascending and descending sounds | Exercise felt easier with the “High” vs “Low” pitch |
| | | Comfort | Exercise felt more comfortable with ascending vs descending sound | - The “Musical” sounds maximize the differences between ascending and descending sounds<br>- Higher feeling of comfort with the “musical” than with the “tone” sounds | Not affected |
| | | Capability | More capable with ascending vs descending sound | The “Musical” sounds maximize the differences between ascending and descending sounds | Not affected |
| | | Coordination | Movement felt more coordinated with the ascending vs descending sound | Not assessed | Sound Direction and Pitch interaction (not significant): The “Musical_down_Low_ pitch” led to feelings of less coordinated movement |
| | Emotional feelings | Motivation | Feeling more motivated with ascending vs descending sound | The “Musical” sounds maximize the differences between ascending and descending sounds | Feeling more motivated with the “High” vs “Low” pitch |
| | | Happiness | Feeling happier with the ascending vs descending sound | Not assessed | Feeling happier with the “High” vs “Low” pitch |
| | | Arousal | Feeling considerably happ ier with the ascending vs descending sound (not significant) | Not assessed | Feeling considerably more excited with the “High” vs “Low” pitch (not significant) |

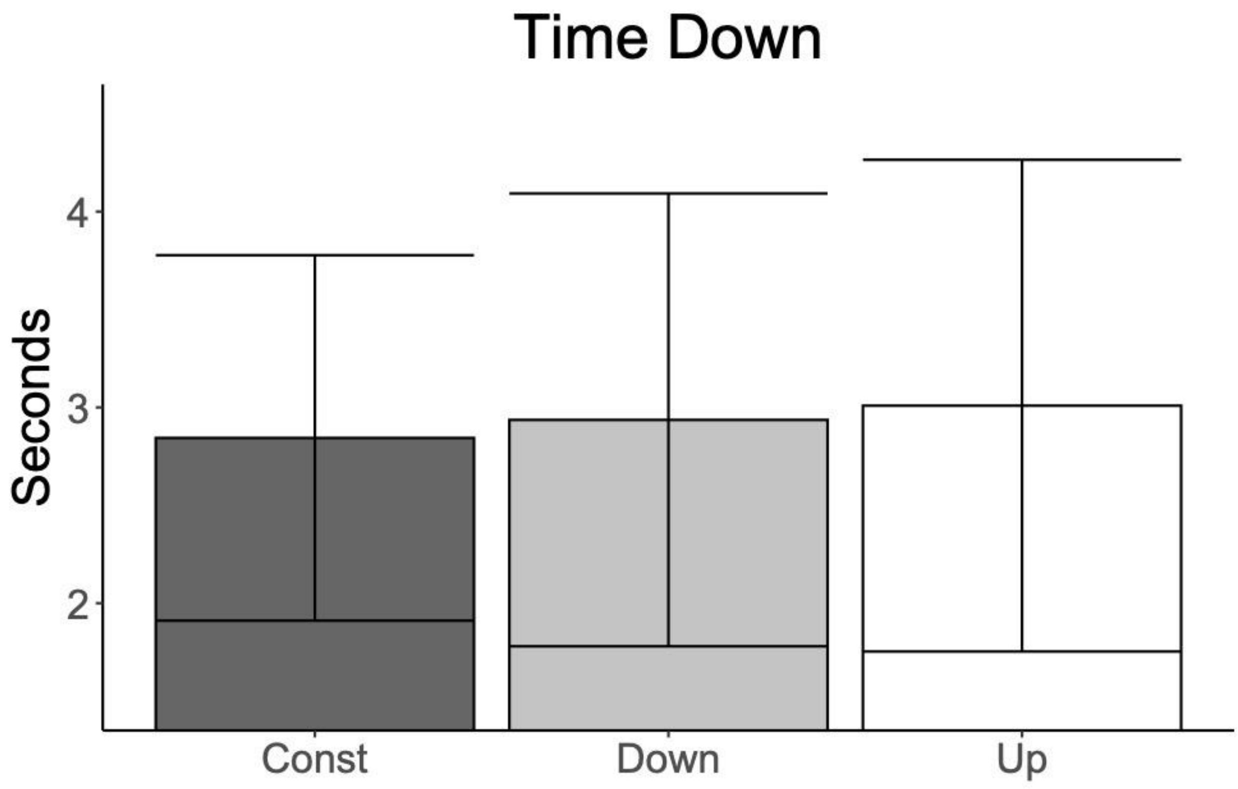


**Figure S1.** Barplot with mean and SD for the parameter “Time down” (seconds), for all sound conditions in Experiment 1.

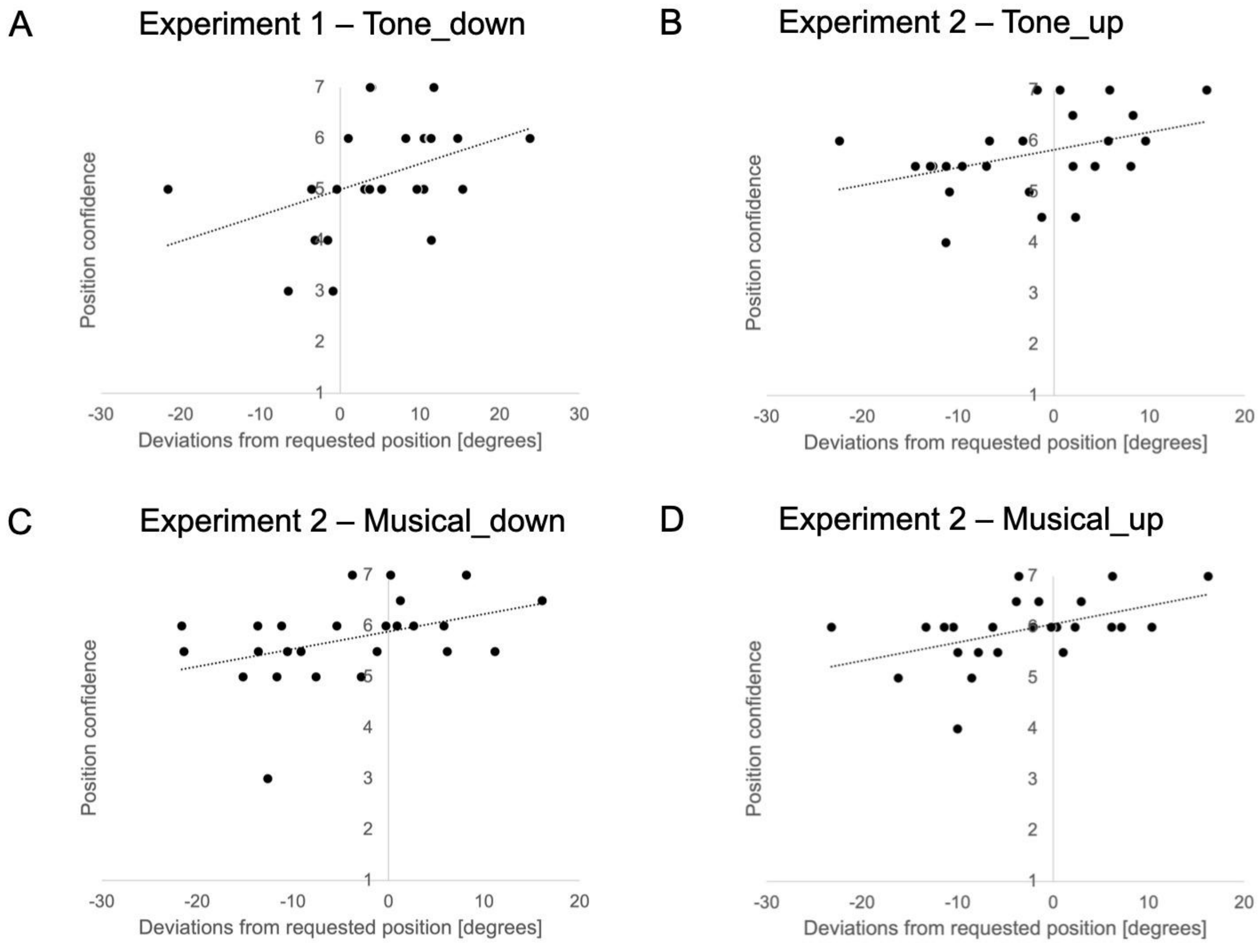


**Figure S2.** Correlations between self-reported confidence in having reached the requested position and actual task performance (angle deviations from the requested positions) for (A) “Tone_down” and Position 1 in Experiment 1, and (B) “Tone-up”, (C) “Musical_down” and (D) “Musical_up” (both Positions) in Experiment 2.

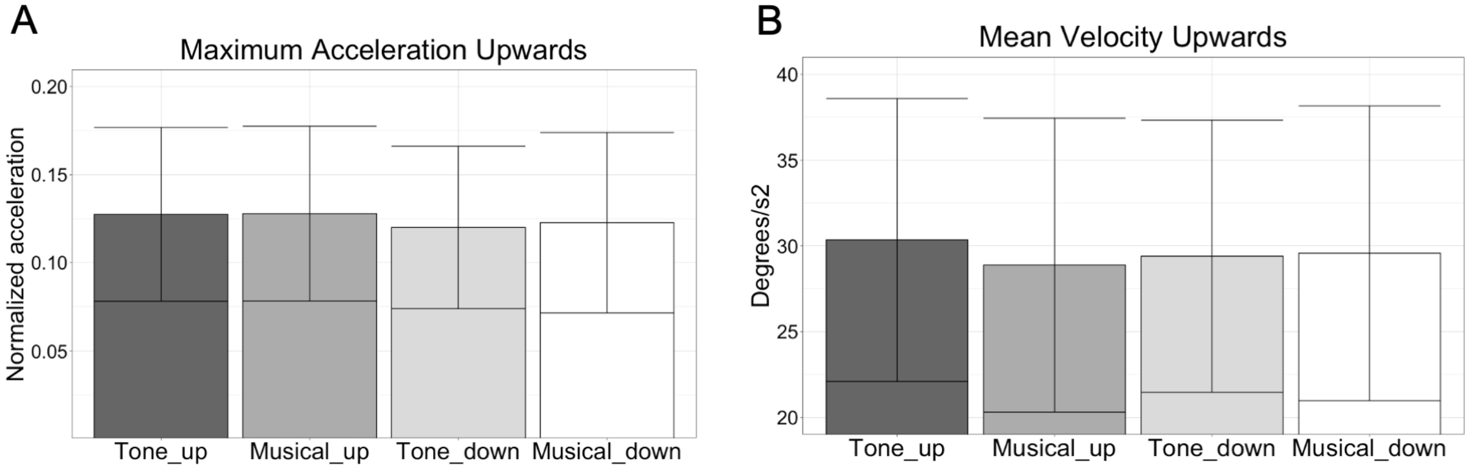


**Figure S3.** Bar plot with mean and SD for acceleration and velocity of the upwards movement in Experiment 2. (a) Acceleration in upwards movement for all Sound Conditions in Position 1, and (b) velocity in upwards movement for all Sound Conditions in Position 1.

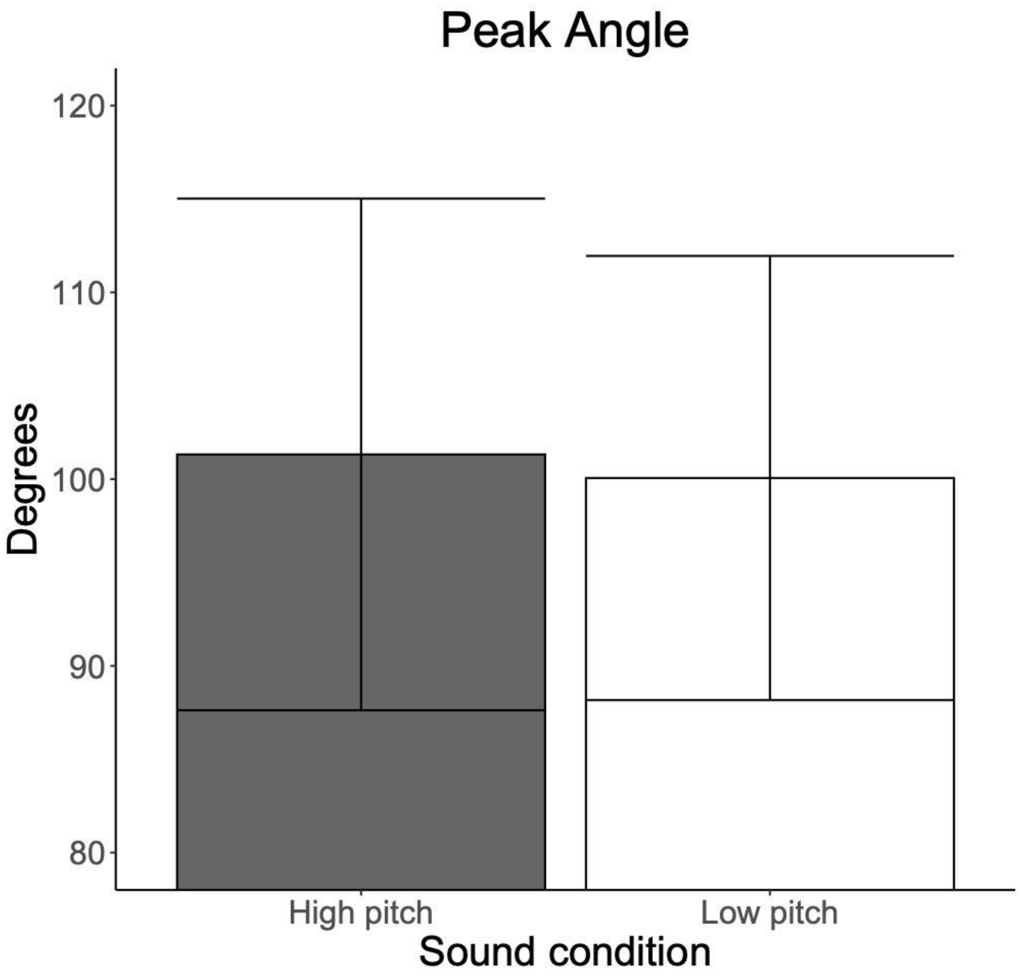


**Figure S4.** Bar chart showing the significant effect of the factor Sound Frequency Range on the maximum reached angle (Peak Angle) in Experiment 3.

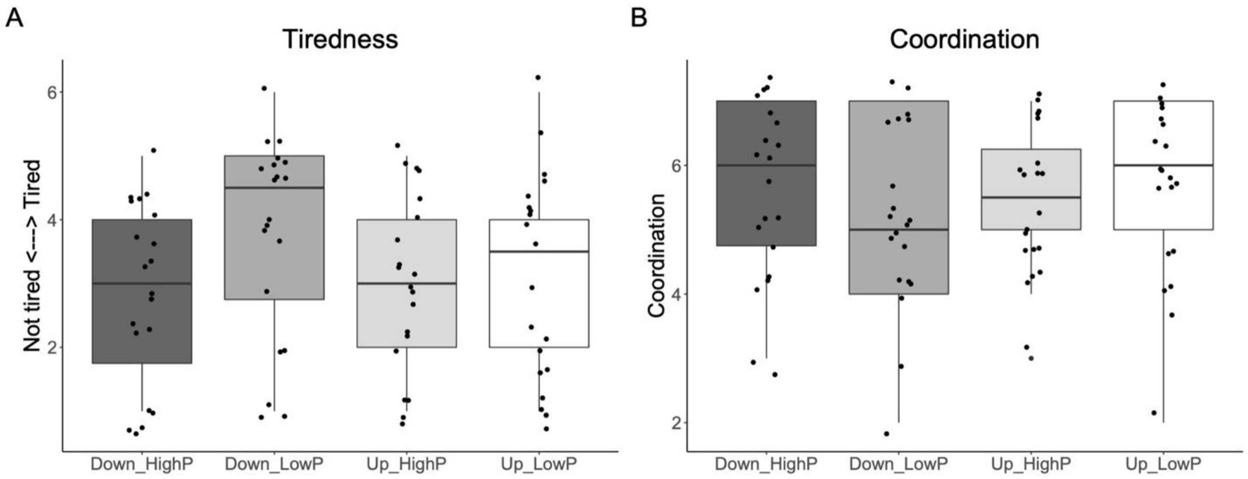


**Figure S5.** Boxplots with median(range) score for questionnaire items showing interaction effects between Sound Direction and Sound Frequency Range in Experiment 3. (A) Feelings of tiredness and (B) movement coordination. Down_High_P = Musical_down_High_pitch, Down_Low_P = Musical_down_Low_pitch, Up_High_P = Musical_up_High_pitch, Up_Low_P = Musical_up_Low_pitch.
